# A Landmark-Free Method for Three-Dimensional Shape Analysis

**DOI:** 10.1371/journal.pone.0150368

**Published:** 2016-03-08

**Authors:** Benjamin J. Pomidor, Jana Makedonska, Dennis E. Slice

**Affiliations:** 1 Department of Scientific Computing, Florida State University, Tallahassee, Florida, United States of America; 2 Department of Anthropology, State University of New York at Albany, Albany, New York, United States of America; 3 Department of Anthropology, University of Vienna, Vienna, Austria; University of Naples, ITALY

## Abstract

**Background:**

The tools and techniques used in morphometrics have always aimed to transform the physical shape of an object into a concise set of numerical data for mathematical analysis. The advent of landmark-based morphometrics opened new avenues of research, but these methods are not without drawbacks. The time investment required of trained individuals to accurately landmark a data set is significant, and the reliance on readily-identifiable physical features can hamper research efforts. This is especially true of those investigating smooth or featureless surfaces.

**Methods:**

In this paper, we present a new method to perform this transformation for data obtained from high-resolution scanning technology. This method uses surface scans, instead of landmarks, to calculate a shape difference metric analogous to Procrustes distance and perform superimposition. This is accomplished by building upon and extending the Iterative Closest Point algorithm. We also explore some new ways this data can be used; for example, we can calculate an averaged surface directly and visualize point-wise shape information over this surface. Finally, we briefly demonstrate this method on a set of primate skulls and compare the results of the new methodology with traditional geometric morphometric analysis.

## Introduction

This paper introduces a novel method, Generalized Procrustes Surface Analysis (GPSA). The method adapts the Iterative Closest Point (ICP) family of algorithms [1][2] to the Generalized Procrustes Analysis (GPA) [3] paradigm. The purpose of GPSA is to use the scanning technology available to researchers today to perform automated, landmark-free shape analysis. By extensively modifying and combining these two algorithms, we can perform a symmetric superimposition of multiple surfaces then calculate a distance metric based on this superimposition.

We use the ICP algorithm for superimposition because surfaces lack the pointwise homology of landmarks, which is needed for methods like Procrustes superimposition. The ICP algorithm approximates this homology by associating each point on one surface with its nearest neighbor on another surface. It then calculates and applies a transformation to minimize a cost function based on the distances between these point pairs. This process is repeated until the distance between surfaces cannot be significantly reduced through additional iteration.

For real world data sets, there are typically more than two surfaces. The ICP algorithm works on pairs of surfaces, much like Procrustes superimposition. To superimpose a collection of surfaces, we take a similar approach to GPA: we designate an estimated mean surface and then superimpose each surface to it. The mean surface is recalculated according to its point associations and the process is repeated. This continues until the changes made to the mean surface are sufficiently small.

With superimposition and homology established, we proceed to the calculation of a distance metric. We cannot use traditional Procrustes distance because of the variable number of points in different surface scans. We instead define a new distance, the Procrustes Surface Metric (PSM). This uses points on both surfaces and weights them appropriately to ensure symmetry. This metric is very closely related to Procrustes distance, and may be used in further analysis in a similar manner.

Development of the method was done in Java, in preparation for eventual integration into the Morpheus et al. geometric morphometrics software package [4]. Morpheus itself was used over the course of method validation, as was the NTSys data format [5]. The Apache Math Commons library was used for linear algebra support [6], while MeshLab was used for additional visualization [7]. High-resolution surface scans of primate skulls were used for testing, demonstration, and validation. The 3D surface scans were generated using a 3D desktop laser scanner model NextEngine [8]. Landmarks were collected from the virtual models using the Landmark Editor software [9]. Free, cross-platform software implementing these superimposition, ordination, and visualization methods, GPSA, is available for download from the software page at our lab website (http://morphlab.sc.fsu.edu). No significant biological interpretation of these results was performed as this paper is focused solely on the presentation of the new method.

## Methods

### Symmetric Iterative Closest Point Superimposition

The primary limitation of the classic ICP algorithm for morphometric analysis is that it is not symmetric. The superimposition of one surface onto another will not necessarily produce a similar superimposition if the order of superimposition is reversed. In classic ICP, a mean squared error cost function is created based on nearest-neighbor pairings from the first surface to the second. The function is then solved for transformation parameters to be applied to the first surface. Here, *C*_*A*_ is the cost function for a basic point-to-point ICP algorithm, *p*_*A*,*i*_ and *q*_*B*,*i*_ are a point on surface A and its nearest neighbor on surface B, *m*_*A*_ is the number of points on surface A and *H* and *t* are rotation and translation terms:

$$\begin{array}{c} C_{A}=\sum_{i=1}^{m_{A}}{∥p_{A,i}-\left(Hq_{B,i}-t\right)∥}^{2} \end{array}$$ (1)

In our implementation, we chose the popular point-to-plane framework [2], which differs by including the normal vector, *n*, in the cost function in order to minimize the distance between one point and the plane on which its paired point lies (see S1 Appendix for more detail). To make this a symmetrical operation, we take both the nearest neighbor pairings for the first surface and the second surface into account for our cost function, which is still written in terms of a transformation applied to a single surface. This is a similar process to the one presented by Godin [10], but derived for a different purpose and framework.

$$\begin{array}{c} C=\sum_{i=1}^{m_{A}}{\left(\left(p_{A,i}-\left(Hq_{B,i}-t\right)\right)\cdot n_{A,i}\right)}^{2}+\sum_{j=1}^{m_{B}}{\left(\left(\left(H^{T}p_{B,j}+t\right)-q_{A,j}\right)\cdot n_{B,j}\right)}^{2} \end{array}$$ (2)

We may linearize *H*, solve the resulting linear system, and then apply the transformation [2][11][12] to surface A. The resulting superimposition minimizes the summed distances to nearest neighbors for all points over both surfaces and is geometrically identical (for the surfaces relative to one another) regardless of which surface is transformed and which remains stationary.

Note, the ICP algorithm is very dependent on the initial positions of the surfaces relative to one another. A good, anatomically consistent initialization method is key to a good superimposition. Our most effective technique was to align the longest and second longest axes of the two surfaces. These were found simply by calculating the first two principal components of the set of 3D points making up each scan. To account for potential reflection of axes, the surfaces were aligned in all possible combinations of reflections. The combination with the lowest calculated PSM value between the surfaces was assumed to be the best alignment and used for initialization.

### Procrustes Surface Metric

To quantify the shape difference between the two surfaces, we use the Procrustes surface metric (PSM), which uses the nearest neighbor pairings from the final superimposition to define the homology between surfaces. While the superimpositions obtained using this modified point-to-plane ICP cost function do not directly minimize point-to-point distance, it is much more in the spirit of the Procrustes distance to use point-to-point distances when calculating this metric. Again considering surfaces A and B, where *p* and *q* represent a point and its nearest neighbor and *m* is the number of points on the surface, this metric is:

$$\begin{array}{c} D=\sqrt{\frac{1}{2m_{A}}\sum_{i=1}^{m_{A}}{\left(p_{A,i}-q_{B,i}\right)}^{2}+\frac{1}{2m_{B}}\sum_{j=1}^{m_{B}}{\left(p_{B,j}-q_{A,j}\right)}^{2}} \end{array}$$ (3)

This metric is similar to a root-mean-squared distance, but it uses the points from both surfaces and weights each surface appropriately so that they each contribute equally to the metric. This metric is accordingly very closely related to Procrustes distance. Given a pair of objects with *m* landmarks, and Procrustes distance *P*, this relation is simply:

$$\begin{array}{c} D=\sqrt{\frac{1}{m}}\cdot P \end{array}$$ (4)

### Prototypes

Symmetric superimposition and shape difference measurement only allow us to analyze a pair of surfaces at a time. To extend the analytical capability of the method and avoid performing pairwise superimposition for all individuals in the sample, we follow GPA and instead superimpose the individuals in a data set to a designated mean or ‘prototype’ surface. This surface should be the most complete, most representative, least morphometrically atypical individual in the sample, as far as the researcher can determine. The reasons for this are discussed later in this section. Once all surfaces in the sample have been superimposed this way, we calculate a new position for each point making up the prototype surface. By finding the nearest-neighbor point on each specimen surface for a given point on the prototype surface, we can calculate an average location of these points. Our approach is similar to the one taken by Toldo et al [13], where they apply a GPA-type method to ICP in order to reconstruct a single surface based on multiple incomplete range images, but is distinct in that they only use mutual nearest neighbors to reconstruct their mean surface. This would not be appropriate in a morphometric context as the surface scans do not all come from a single object where an exact correspondence would be expected. Instead, we use the entire nearest neighbor set with no exclusions.

This average location is the new position assigned to the point on the prototype surface. Once all points on the prototype have been assigned a new position, any identical points (from identical sets of nearest neighbors) are removed from the prototype to prevent overweighting a particular region. To do this, one can use a hash function and sort or radix-like search. This new prototype is then used for a further full-sample superimposition pass (see S1 Appendix for an outline of our duplicate point elimination method). This process, like in GPA, is repeated until the change in the shape of the prototype/mean surface is negligible.

Note that through this process, any surface used as the prototype will be reformed to fit the final distribution of points in the set of superimposed surfaces. If the initial prototype surface is incomplete or not representative of physical features found in the set, the points making up the surface will still be transformed to fit the mean of the distribution of their nearest neighbors in the superimposed data set. However, these poorly represented regions will continue to be poorly represented throughout the superimposition process and will ultimately be missing or malformed on the final prototype surface. For samples with high variation, like the multi-species case demonstrated later in this paper, it is less likely that the nearest neighbor pairings will represent an accurate homology. This means that the shape of the final prototype becomes more dependent on the original prototype as variation in the sample increases. Consequently, it is important that the researcher exercise appropriate judgment when selecting the initial prototype surface.

In practice, more than three iterations did not appreciably change the shape of the prototype surface for our data. This may be dependent on the data set, so care should be taken to ensure the shape is stable. It should be noted that the nearest neighbor search in the ICP algorithm is computationally expensive, so use of some kind of space partitioning tree is helpful [14]. In our implementation, we used a k-means based binary space partitioning tree [15] to good effect (see S1 Appendix for an overview of the BSP tree we used).

### Method Summary

A summary of the GPSA method follows:

1. Select prototype and set initial surface positions
2. For each surface in the sample:
  - Superimpose via symmetric ICP to the prototype surface
  - Store prototype surface → sample surface point pairings
3. Use prototype pairings to update averaged positions for individual points on the prototype surface and remove duplicate points
4. Use Procrustes surface metric to measure the difference between the old prototype and the new prototype
  - If distance is small or enough iterations have been completed, terminate the algorithm
  - Otherwise, repeat steps 2 through 4
5. Use Procrustes surface metric to quantify difference between superimposed surfaces

## Results

**Fig 1:**
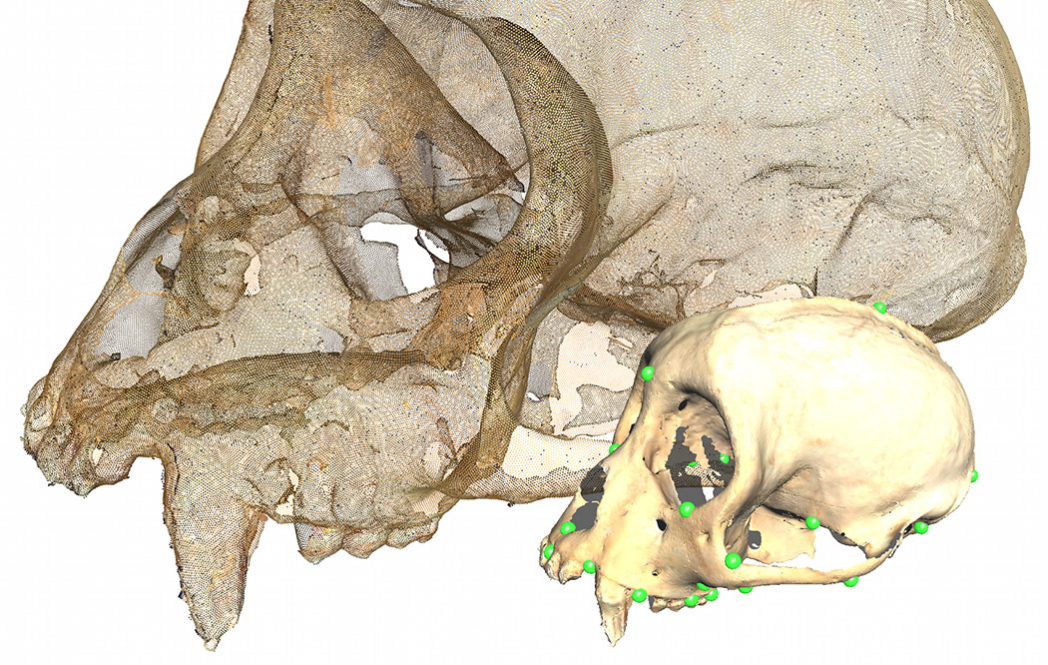
Landmark and surface point distributions for a single specimen. Foreground: A single specimen with the landmarks highlighted. Background: The same specimen rendered only as the points making up the surface. Note the relatively high density of points in the surface scan and the uneven distribution of landmarks.

To test and validate this method, 3D surface scans and landmark data taken from primate skulls were passed through both GPA and GPSA, with size removed and size restored. The data consisted of two samples: one composed of fifteen adult male *Cebus spp.* and the other composed of eighteen adult male (three specimens per genus) *Cebus spp.*, *Chiropotes spp.*, *Colobus spp.*, *Papio spp.*, *Piliocolobus spp.*, and *Pithecia spp*‥ The first sample was used to represent a relatively homogeneous data set with limited variation. The second was used to evaluate the performance of the method in the face of relatively extreme shape variation. These samples were tested both with and without size as a factor (by scaling to unit centroid size). The selected osteometric landmarks could be reliably identified on all models in both samples and consisted of forty-three landmarks predominantly distributed on the ventral side of the skull, though some neurocranial and facial landmarks are included (Fig 1). Each generated virtual surface represents a mesh composed of hundreds of thousands of triangles drawn between hundreds of thousands of 3D coordinates. These surface scans contained anywhere from approximately 300,000 to 1,000,000 points. Due to shape and size diversity, specimens belonging to different species required slightly different scanning modes. Small, five-to-fifteen-centimeter skulls were placed closer to the scanner and scanned with a precision of 0.005 inches and a resolution of 10,000 points per square inch, while large, baboon-sized skulls had to be placed relatively far from the scanner, and were scanned with an accuracy of 0.015 inches and a resolution of 1,100 points per square inch.

Two aspects of GPSA were evaluated: the quality of the superimposition and the practical similarity of the Procrustes surface metric (PSM) to Procrustes distance. We did this by superimposing the data using both GPSA and GPA, then measuring shape difference pairwise between individuals in a sample using both Procrustes distance and PSM. This effectively gives us four sets of interspecimen distances for a given data set: Procrustes distance for GPA superimposition, PSM for GPA superimposition, Procrustes distance for GPSA superimposition, and PSM for GPSA superimposition. We can then plot these distances against one another to understand the relationship between the superimposition methods and shape-difference measurement metrics.

**Fig 2:**
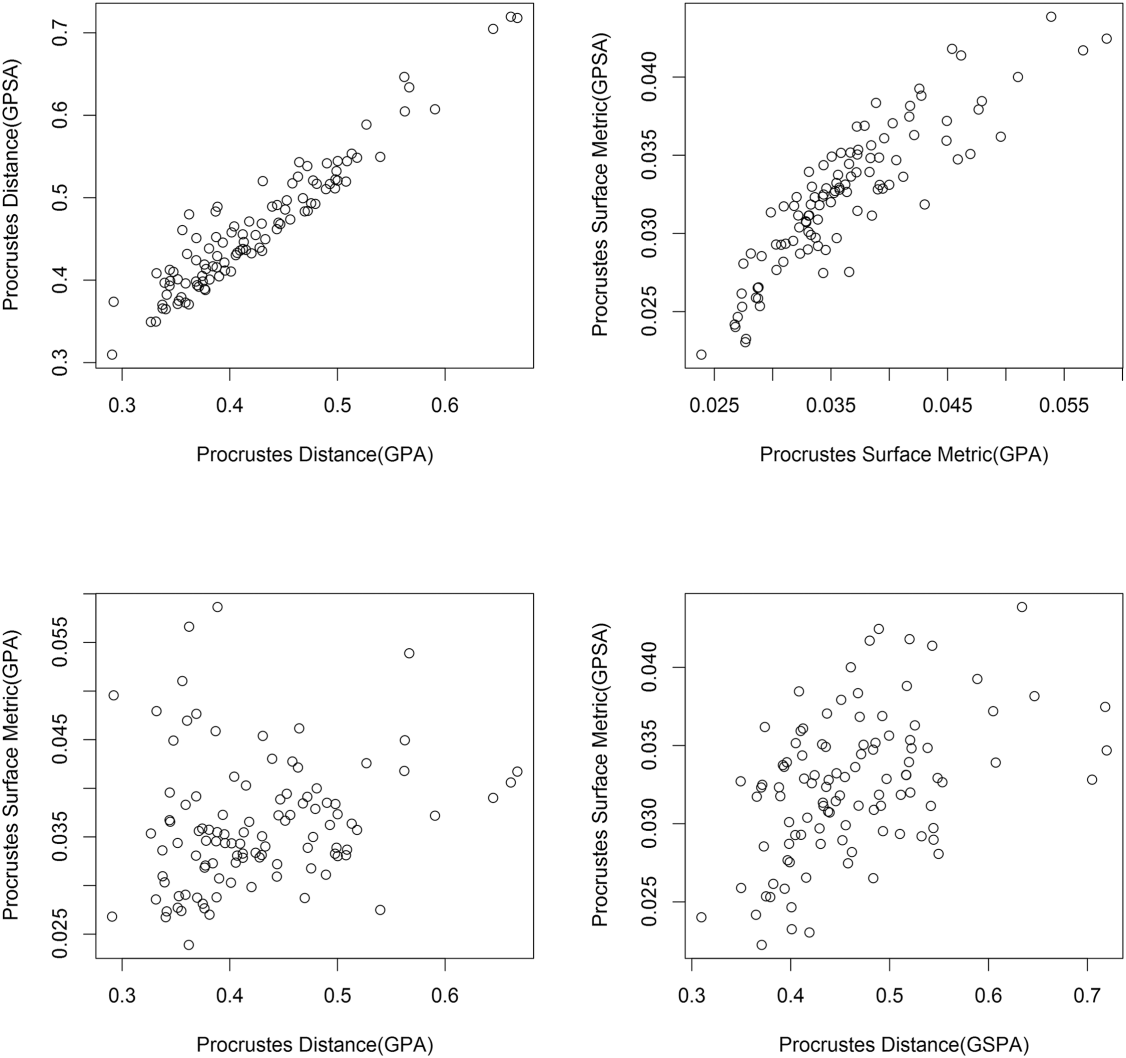
Graphs comparing aspects of GPA and GPSA for the single species sample with size removed. Top left: GPSA and GPA superimpositions compared using Procrustes distance. Note the high correlation. Top right: The two superimpositions compared using Procrustes surface metric. Note the high correlation. Bottom left: Procrustes surface metric and Procrustes distance measures compared using GPA superimposition. Note the low correlation. Bottom right: The shape distance measures compared using GPSA superimposition. Note the low correlation.

After performing the above procedure on the single species data set with size restored, we can compare the two superimpositions by plotting the Procrustes distances for each superimposition against one another (Fig 2, top left). This plot yields a fairly good correlation. Repeating this test with PSM values instead of Procrustes distance also yields a slightly poorer correlation (Fig 2, top right). This indicates that there is little change in relative superimposition for this data set, at least as measured by PSM and Procrustes distance. Also, the slight difference in correlation is evidence that PSM is more sensitive to this small change in superimposition than Procrustes distance. Note the range of values in the two plots: as one would expect, the GPA superimposition more successfully minimized Procrustes distance and the GPSA superimposition more successfully minimized PSM.

To compare the shape difference measurements, we superimpose the data set using GPA, then again plot the corresponding pairwise specimen distances against one another, this time for Procrustes distance and PSM values (Fig 2, bottom left). There is virtually no correlation between the two. Repeating the plot for the GPSA superimposition, we see a weak but positive correlation (Fig 2, bottom right). This increase in correlation reinforces the notion that PSM is more sensitive than Procrustes distance to the difference in superimposition as the GPSA superimposition had the better correlation. The similarity of two superimpositions methods taken together with the poor correlation between the two metrics for a given superimposition is evidence of a wide difference in the shape information captured by the two shape difference metrics.

**Fig 3:**
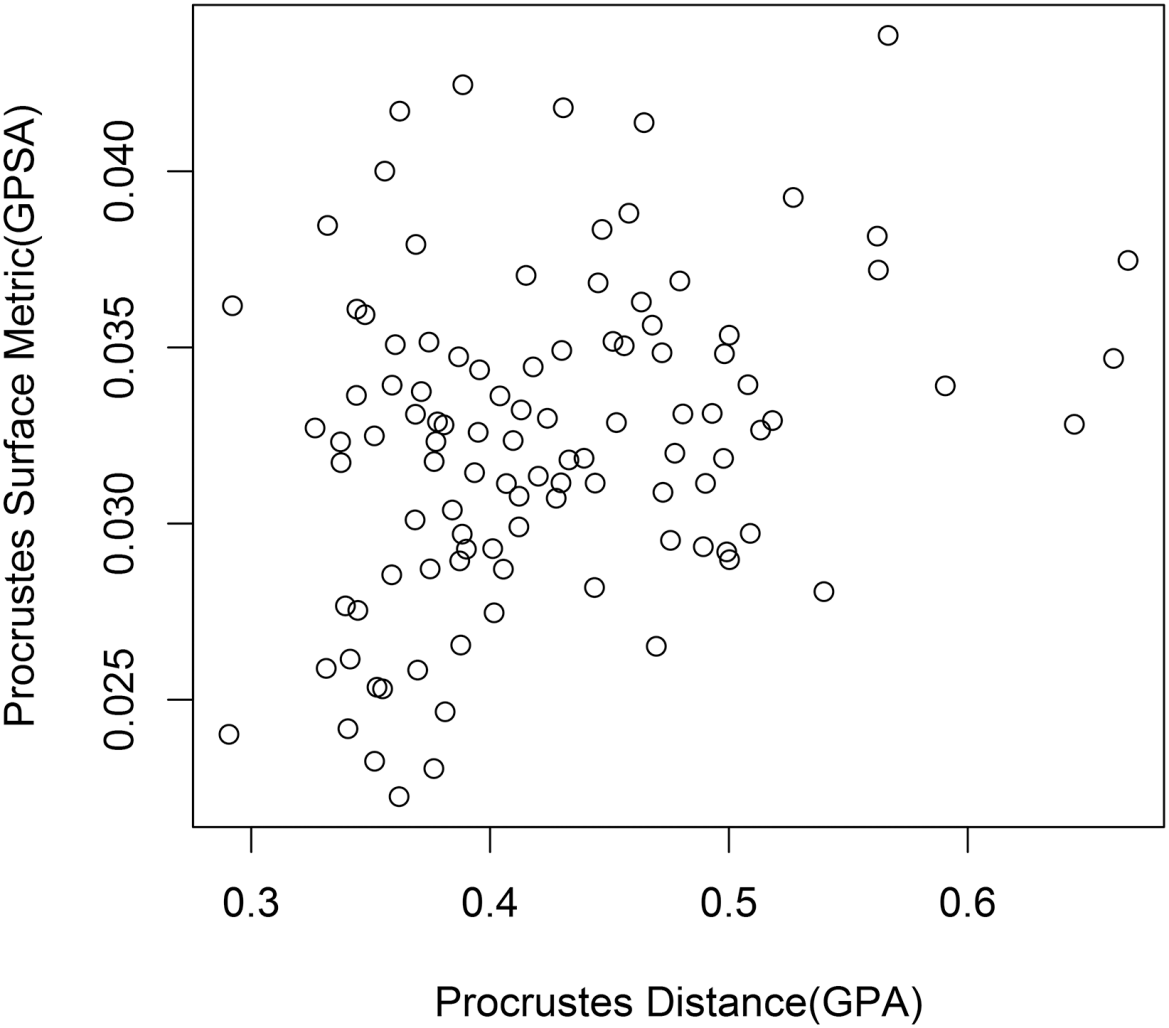
Pairwise interspecimen shape distances from the single species sample with size removed. The interspecimen Procrustes surface metric values for a GPSA superimposition are plotted against the interspecimen Procrustes distance values for a GPA superimposition. There is a slight positive correlation.

**Fig 4:**
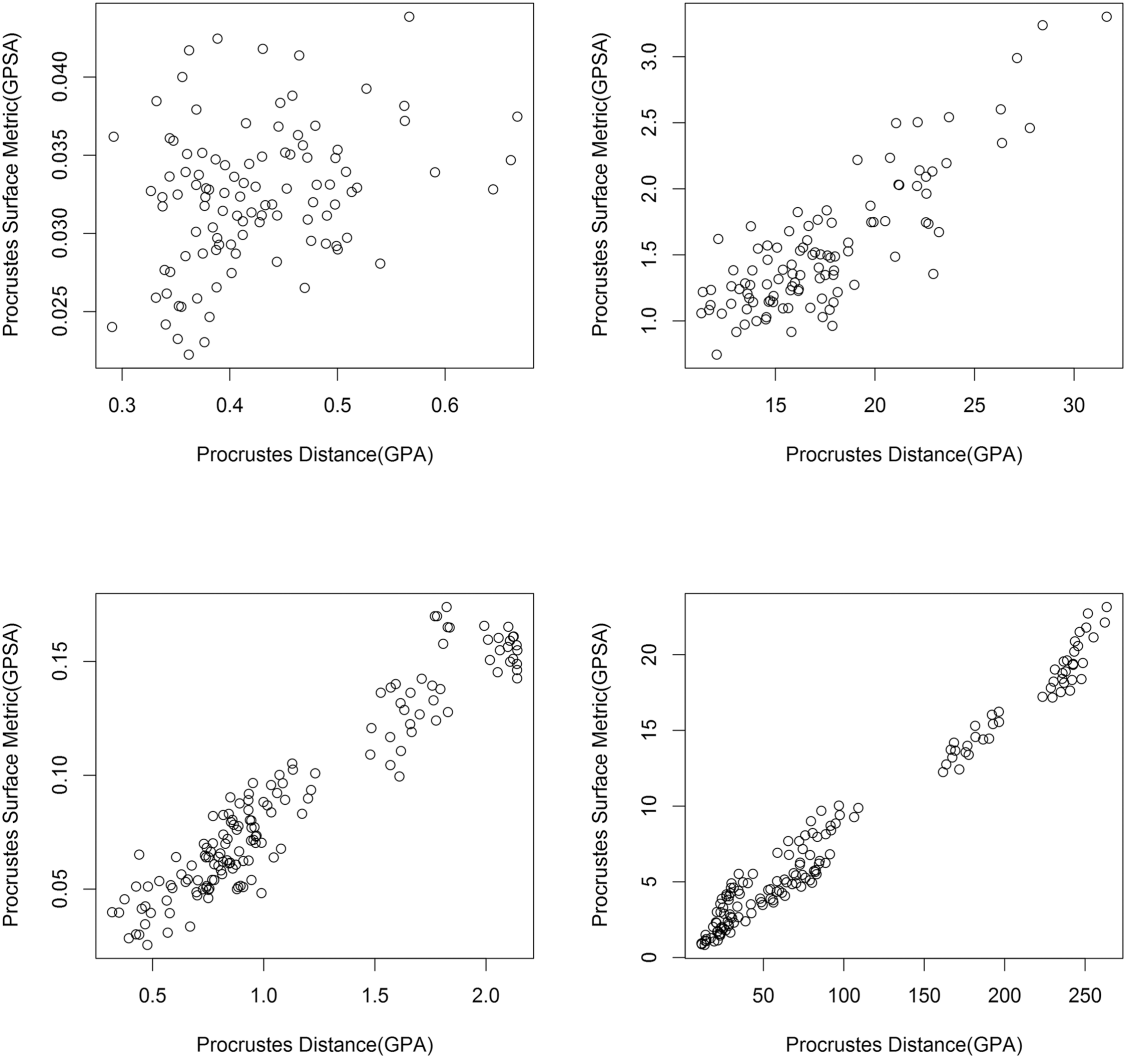
Graphs comparing GPSA to GPA for the two samples, with and without size. Top left: The single species sample with size removed. Top right: The single species sample with size restored. Bottom left: The multiple species sample with size removed. Bottom right: The multiple species sample size restored. As expected, the two methods become more correlated with increasing shape and form variability.

Finally, we can plot the two methods as they would be used in practice: a GPSA superimposition with PSM measurements against a GPA superimposition with Procrustes distance measurements (Fig 3). The resulting graph shows a very weak positive correlation. The general characteristics of the single species sample with size removed generally hold whether or not multiple species or size are included. However, with the addition of such influential shape or form information, there is an increase in the correlation between the two methods, visible when we plot this final graph for each data set in the series (Fig 4).

**Fig 5:**
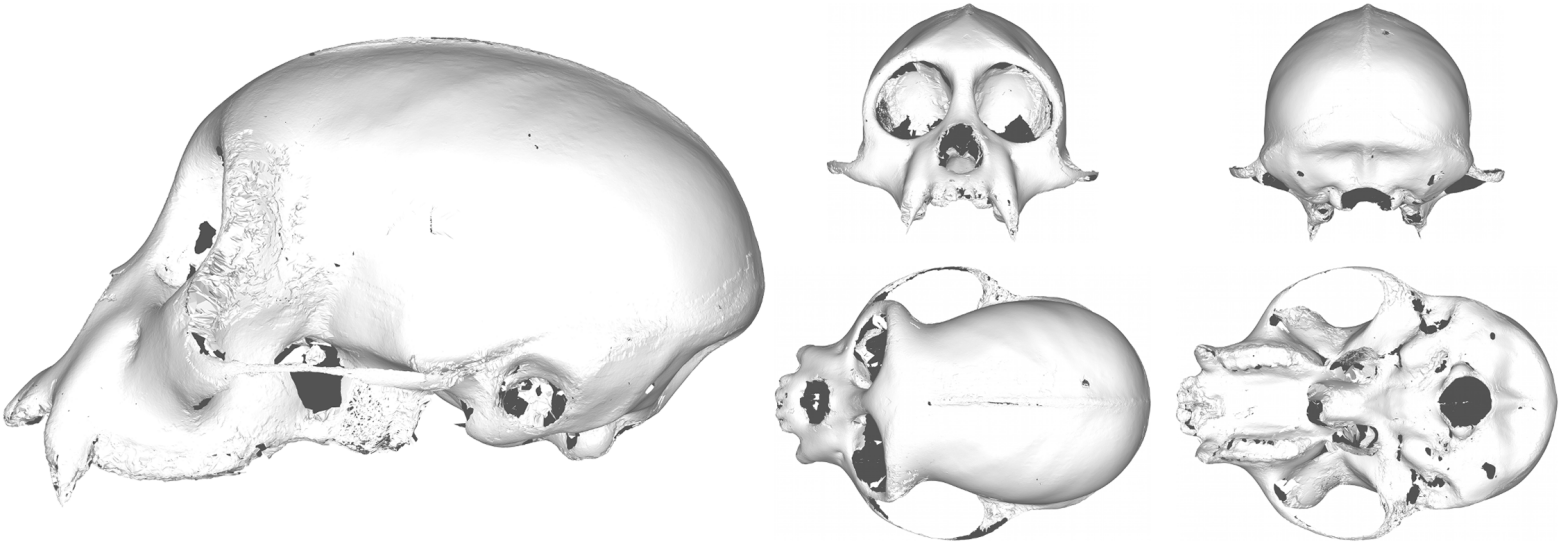
The final prototype surface for the single species sample with size removed. This surface is the calculated average surface to which all surfaces in the sample are superimposed over the course of GPSA. Note the reduction in size of the zygomatic arch due to surface collapse.

**Fig 6:**
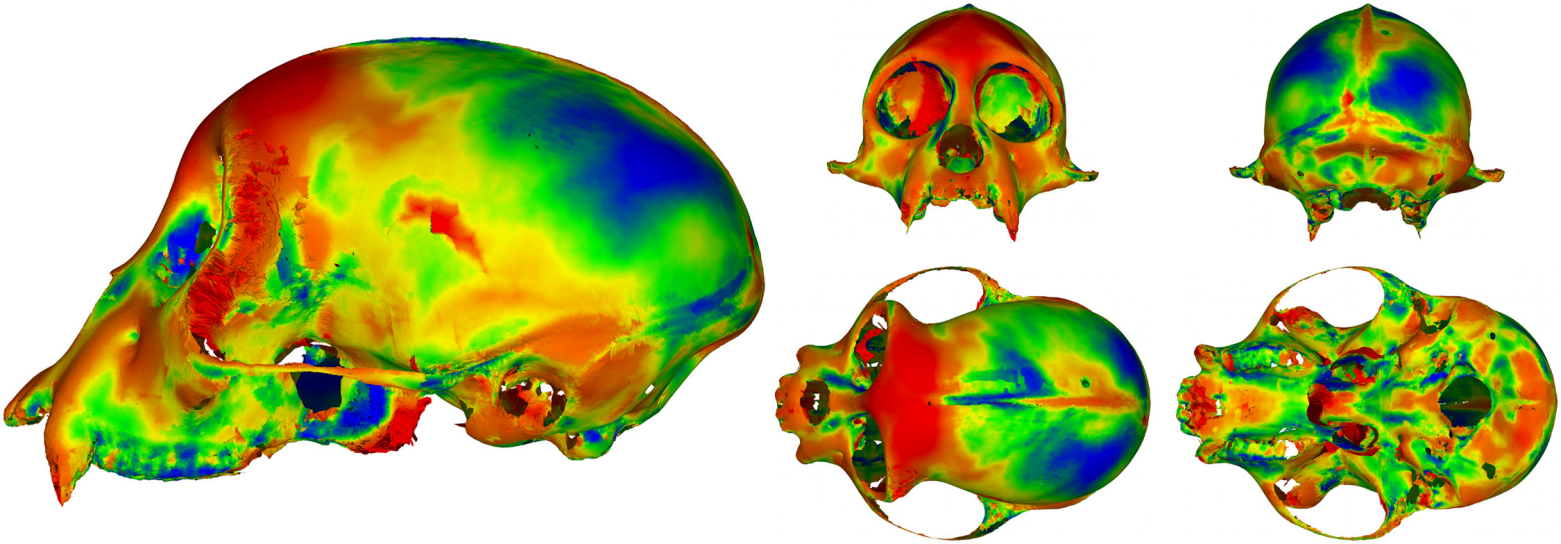
A heat map of the variance from the prototype for the single species sample with size removed. Blue indicates low values, red indicates high values. These values are calculated from the covariance matrix of the set of nearest neighbor points for each point on the prototype. Note the high variance region in the center of the neurocranial region in the side view. One specimen had a large hole in this region, which is reflected in the heat map.

**Fig 7:**
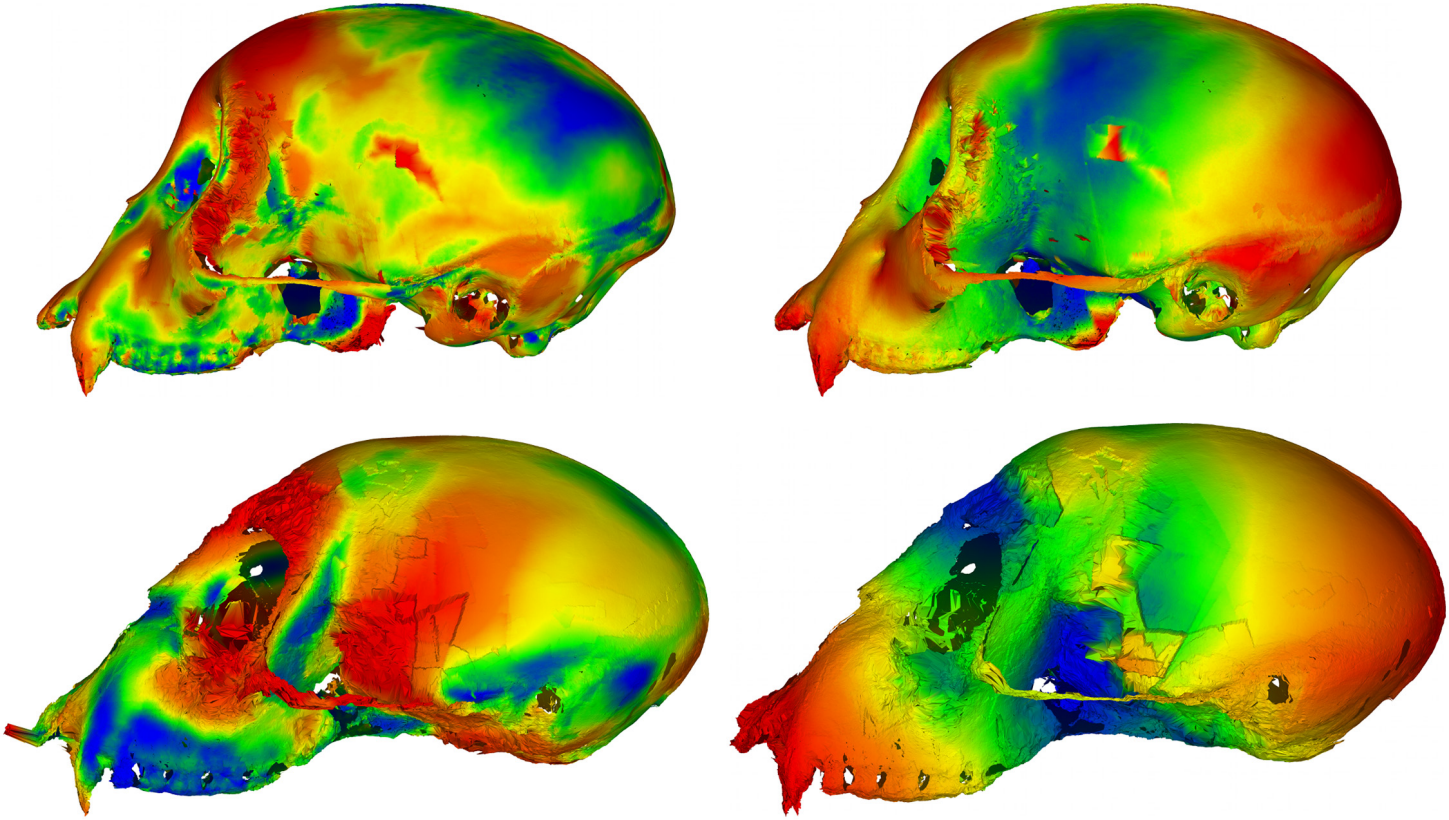
The heat maps of the two samples, with and without size. Blue indicates low values, red indicates high values. Top left: The single species sample with size removed. Top right: The single species sample with size restored. Bottom left: The multiple species sample with size removed. Bottom right: The multiple species sample size restored.

One major advantage of performing analysis in a point-wise manner over an entire surface, rather than on a small set of hand-selected landmarks, is the large amount of data available to the researcher post-analysis. After superimposing the data, we have the entire mean surface available as the prototype, with no extra calculation(Fig 5). We are able to calculate the variance at every point on the prototype. This pointwise variance can then be mapped to colors and attached to the prototype, effectively creating a three dimensional heat map representative of the sample variation(Figs 6 and 7).

## Statistical Analysis

Vertex coordinates homologized and superimposed by GPSA can then be used to address project-specific, multivariate statistical questions. This might include appropriate tests for group differences or regression of shape onto size, age, mean temperature or other factor. One of the most frequently used multivariate techniques is ordination and dimension reduction. Ordination techniques, such a principal components analysis (PCA), create variables that are composites of the original variables to emphasize some attribute of the full data set. In PCA, this is a linear combination of the original variables (coordinates) that are orthogonal basis vectors of the multivariate data space ordered by the magnitude of variance accounted for in the data. A smaller subset of these might be selected as the foundation for further analysis as a lower-dimensional, variance-maximizing subset of the original full data set. The extremely high dimensionality of vertex data, however, might cause computational difficulties. One way to address this is described in more detail in S1 Appendix.

**Fig 8:**
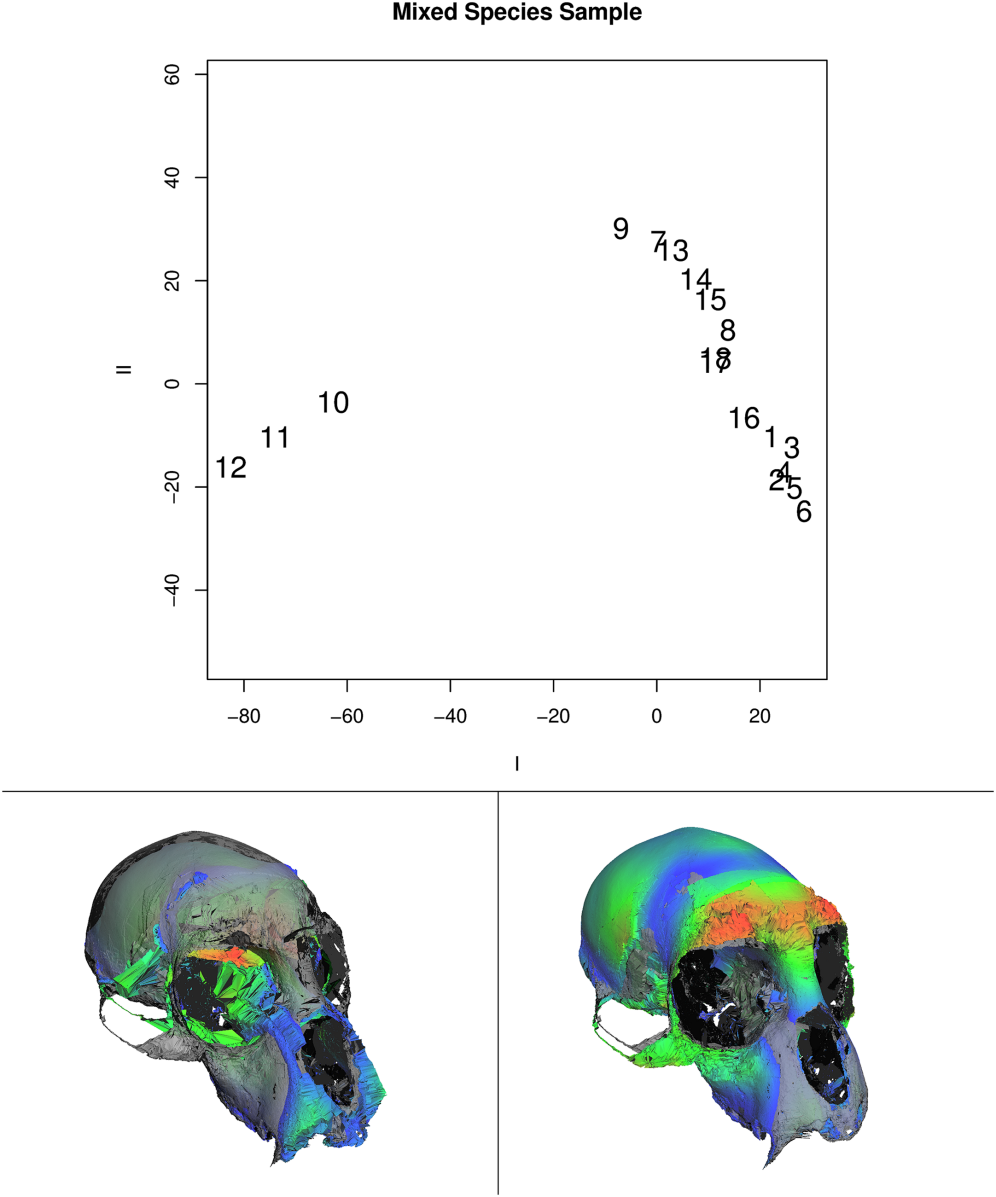
Principal axes of shape variation for the mixed-species data. Top shows the axis of greatest (I) and second greatest (II) variance (see S1 Appendix for details). Lower left panel shows visualization of specimen surface with lowest projection (specimen 12, score ≈ -82.6) on axis I. Lower right panel shows the same for the specimen with the highest projection (specimen 6, score ≈ 28.5). Coloring indicates magnitude of displacements due to the vertex regression coefficients. Mean surface is shown in semi-transparent gray for comparison.

**Fig 9:**
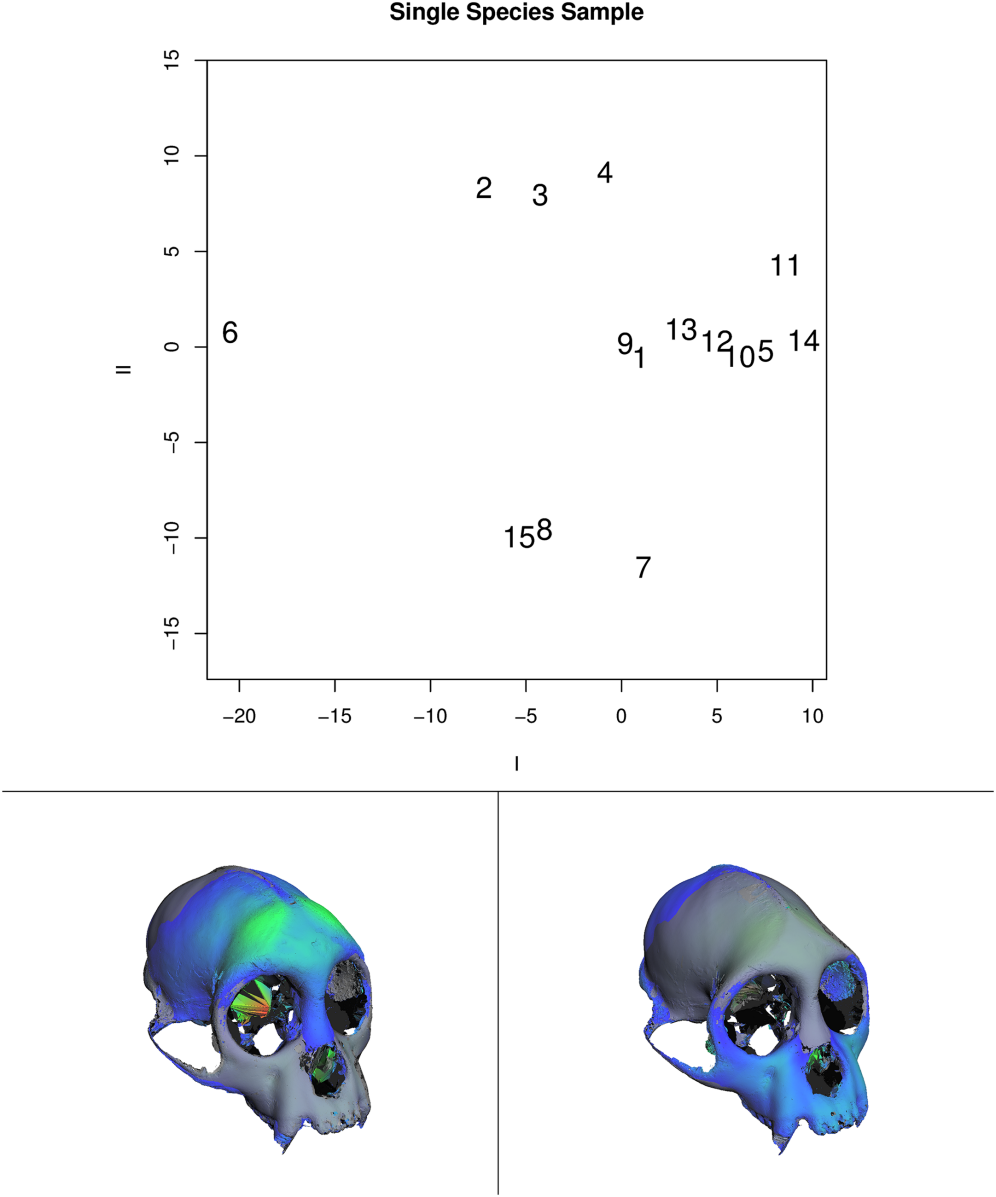
Principal axes of shape variation for the single-species, single-sex data. Top shows the axis of greatest (I) and second greatest (II) variance (see S1 Appendix for details). Lower left panel shows visualization of specimen surface with lowest projection (specimen 6, score ≈ -20.5) on axis I. Lower right panel shows the same for the specimen with the highest projection (specimen 14, score ≈ 9.5). Coloring indicates magnitude of displacements due to the vertex regression coefficients. Mean surface is shown in semi-transparent gray for comparison.

Figs 8 and 9 show examples of such a dimension-reduction technique applied to the example data used here along with the visualizations afforded by GPSA. Fig 8 shows the plot of the first two axes of major variation for the mixed-species data set. Along the axis of greatest variation, specimens 10, 11, and 12 are contrasted with the rest of the sample. These specimens are all members of the genus *Papio*, baboons, which are quite distinctive from the other monkey species in the sample. The panels at the bottom of the figure show visualizations (color) of the minimum (left) and maximum (right) projections of surfaces onto the first axis. These are shown with a transparently rendered mean surface (gray) for contrast. The visualization shows the contrast between the relatively (data were isometrically scaled to a common size) elongated face of the baboons and the relatively larger cranial vault of the other monkeys. Variation in the zygomatic arch is also apparent. The color coding is based on the sum, squared magnitude of regression coefficients at different vertices, and emphasizes the relatively high (orange-red) variation in the brow ridge.

The color-coding also shows the potential contribution of more isolated vertex displacement (back of orbit on the lower left panel) that are mostly due to poor scanning of semi-occluded surfaces in the original scan. One should be concerned that these artifacts could drive the analysis, but the logical interpretability of this axis as the contrast between *Papio* and the other species and the overall consistency and interpretability of the rest of the color mapping suggests this is a minor issue in this example. For this exposition, we did no cleaning or editing of the original scans provided to us. This was intended to represent a “worst-case” scenario to stress the method. In actual application, researchers would be likely to carefully clean individual scans, thus reducing the potential impact of such regions.

Fig 9 shows comparable results and visualizations for the single-species, single-sex sample. Here, as expected, the data is much less structured, though within-species-sex variation still is dominated by relative vault-face variation. Again, possible issues related to semi-occluded surfaces are still apparent as seen in the back of the orbit in the lower left panel and within the nasal aperture in the lower right figure. Since color-coding is scaled from min-to-max sum, squared-vertex regression coefficients, the lack of any orange-red regions indicated they are simply not visible in the current view.

## Discussion

GPSA and its associated distance measurement, the Procrustes surface metric (PSM), make a useful addition to the geometric morphometric toolkit. The combined method provides researchers new avenues for shape analysis and can save significant amounts of time-consuming effort, though the automated superimposition comes with its own challenges in terms of proper initialization and an effective nearest neighbor search. The superimposition achieved by the new method was similar to the one found via landmarks, but the interspecimen PSM values were not strongly correlated with their Procrustes distance counterparts for a given superimposition. However, when paired with the appropriate superimposition method, there was a weak, positive correlation between PSM values and Procrustes distance that increased with increasing shape and form variation.

As previously noted, the similarity in superimposition and low correlation in interspecimen shape distances between GPA and GPSA (for the single species case) is indicative of a difference in the shape information captured by each technique. The increase in correlation with increase in variation (Fig 4) is evidence of this property, as the increased shape and form variation is present in both the landmark and surface data. The large amount of variation effectively eclipses the differences between the two methods present in the lowest variation set, leading to the increase in correlation. This also confirms that GPSA and PSM successfully minimize and measure the difference in shape and that in practice they are related to GPA and Procrustes distance, though far from identical.

**Fig 10:**
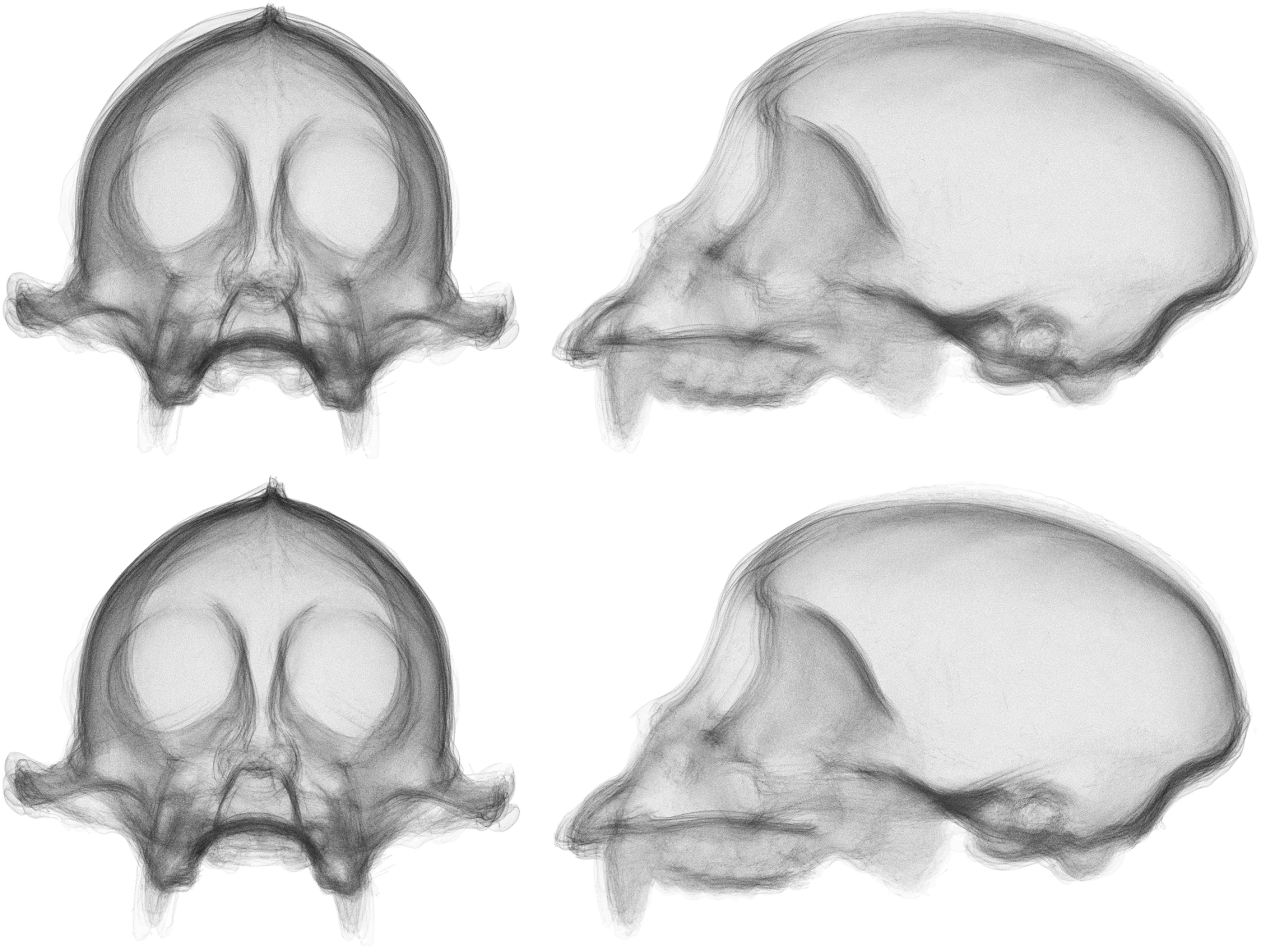
Comparison of superimpositions for the single species sample with size removed. Top: GPA superimposed skulls from the front and side. Bottom: GPSA superimposed skulls from the front and side. Note that there is virtually no visible difference between the two superimpositions.

**Fig 11:**
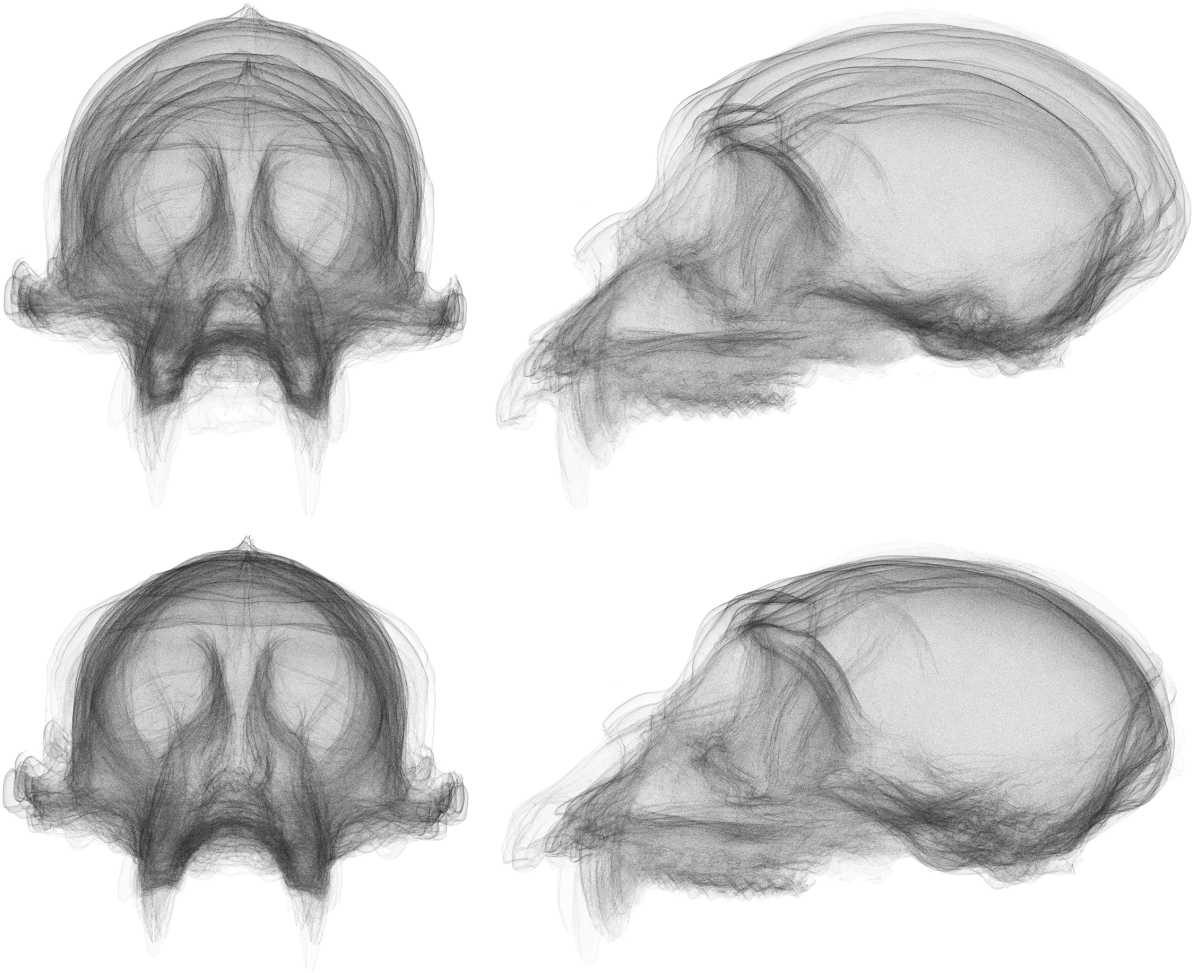
Comparison of superimpositions for the multiple species sample with size removed. Top: GPA superimposed skulls from the front and side. Bottom: GPSA superimposed skulls from the front and side. Note the dramatic difference in superimposition in the neurocranial region.

To an extent, the results of our experiment are to be expected. The distribution of landmarks across the skull is not particularly even, resulting in less emphasis placed on aligning the neurocranial region of the skull when compared to an alignment using the surface scan. This is clear to the naked eye from an image of the superimposed surfaces (Figs 10 and 11). The uneven distribution of landmarks also provides an explanation for the large discrepancy in shape difference measurement. With the landmarks located predominantly on the ventral side of the skull, there is little sensitivity to the vault region, so any shape difference in that region contributes less to the calculated Procrustes distance values. In contrast, the surface-based method uses all available points in the surface scan to perform superimposition and shape-difference measurement. As these points are typically very evenly distributed, it gives the neurocranial region comparatively more weight. There is also the high-resolution nature of the scans themselves, when compared to landmark data sets, which means that any small morphological features that might otherwise be dismissed as textural elements are included in the analysis if they are large enough to be detected by the scanning hardware.

These facets of GPSA mean that researchers using the method must be aware of the distribution of points in their scans to ensure a particular region is not given too much weight. A nonuniform point density may result in a better fit in high-density regions and a poorer fit in low-density regions during superimposition, and lend more weight to shape differences in the high-density region and less weight to shape differences in the low-density region when calculating PSM values. Such a distribution might arise from the application of a decimation algorithm that prioritizes small detail and the simplification of smooth regions. The inclusion of all data on a surface also means that any artifacts from scanning, extraneous anatomy, or missing data (holes) are a part of the analysis, so care must be taken to make sure any such issues are eliminated prior to applying the method.

Another complication with the algorithm in practice is the loss of points in the prototype while it is being reconstructed on each iteration. This occurs as multiple points on the prototype surface are paired with the same set of nearest neighbors on individuals in the sample. When averaged, this places these prototype points in an identical location. While the identical point removal technique previously mentioned works well, we lose information that should be captured by the collapsed surface. An excellent example of this behavior is the zygomatic process in the experimental data (Fig 5). This is mostly an issue in high-variance regions, where edge points are more likely to be close as surfaces will not overlap well, and more complex point matching methods may address the issue in the future. This would also help ensure more points on the sample surface are included in the prototype reconstruction, as only the nearest neighbors to the prototype are currently used.

This method is not necessarily a replacement for landmarks in the morphometric toolkit. While landmark methods are labor-intensive and do not include as much shape information as a surface scan does, this shape information is not always desirable or even biologically relevant. A researcher may only be interested in easily landmarked features, specific regions, or more general shape characteristics. Landmarks have a definite homology, making analysis of high variation data sets much easier. Obtaining clean surface scans is also nontrivial: scanning occlusions and artifacts would affect GPSA superimpositions and PSM values. However, surface-based methods, like GPSA, are able to investigate smooth, featureless regions, do not require the time investment of landmark placement, and allow for unique visualization techniques.

We expect more advanced point-matching methods, accelerated ICP implementations, a unified metric and cost function, parallelization, and better prototype deformation techniques currently in development to improve the speed, accuracy, homology, and usability significantly. For now, GPSA provides a framework for landmark-free superimposition and shape analysis. It unbinds researchers from the constraints of landmarks while simultaneously offering new options for visualization and interpretation. In the Adams et al. review of geometric morphometric methods [16], the authors specifically state that there is a deficiency in visualization of 3D geometric morphometric analysis and further note that full surface analysis could be the next step in the evolution of geometric morphometric methods. GPSA makes significant headway on this path, both using entire surface scans in shape analysis and providing the means to visualize this analysis.

## Supporting Information

S1 Appendix An appendix containing derivations and explanations of methods. This includes a derivation of the symmetric point-to-plane ICP method, a discussion on binary space partitioning trees, an overview of the duplicate point elimination method, and an explanation of the ordination, dimension reduction, and visualization techniques.
(PDF)
