# Supplementary material for: A Landmark-Free Method for Three-Dimensional Shape Analysis
Source: PLoS One. 2016 Mar 8;11(3):e0150368. doi: 10.1371/journal.pone.0150368 (PMC4783062; doi:10.1371/journal.pone.0150368)
Supplement: S1 Appendix — This includes a derivation of the symmetric point-to-plane ICP method, a discussion on binary space partitioning trees, an overview of the duplicate point elimination method, and an explanation of the ordination, dimension reduction, and visualization techniques. (PDF) [file pone.0150368.s001.pdf]

S1 Appendix

1 Symmetric Point to Plane Derivation

The ICP algorithm is a popular method for superimposing range images. It works, at its most basic level, by defining a cost function based on pairs of nearest-neighboring points in terms of transformational parameters. Minimizing this cost function, summed over all points on a surface, gives us transformation parameters that will reduce the total distance between all points on one surface and their nearest neighbors on the other surface. Applying these parameters, then repeating the process will eventually converge to a local minimum, where the surfaces are considered superimposed. Because this is only a local minima, whether this superimposition is actually the best superimposition strongly depends on the initial positioning, making initialization technique very important.

Here, we derive the symmetric point-to-plane formulation used in the GPSA method paper. The derivation presented here is based originally on the one presented by Rusinkiewicz in an unpublished paper [2] and expanded for the symmetric formulation. We begin by performing a linearization of H. First consider $H = H_x \cdot H_y \cdot H_z$, where H_x, H_y, and H_z are the rotation matrices about each axis.

$$H_x = \begin{pmatrix} 1 & 0 & 0 \\ 0 & cos(r_x) & -sin(r_x) \\ 0 & sin(r_x) & cos(r_x) \end{pmatrix} \tag{1a}$$

$$H_y = \begin{pmatrix} cos(r_y) & 0 & sin(r_y) \\ 0 & 1 & 0 \\ -sin(r_y) & 0 & cos(r_y) \end{pmatrix} \tag{1b}$$

$$H_z = \begin{pmatrix} cos(r_z) & -sin(r_z) & 0 \\ sin(r_z) & cos(r_z) & 0 \\ 0 & 0 & 1 \end{pmatrix} \tag{1c}$$

Now, we know that on each iteration, the rotation angles applied should be small. Because of this, we can approximate $cos(r)$ as 1 and $sin(r)$ as r. This gives us:

$$H_x \approx \begin{pmatrix} 1 & 0 & 0 \\ 0 & 1 & -r_x \\ 0 & r_x & 1 \end{pmatrix} \tag{2a}$$

$$H_y \approx \begin{pmatrix} 1 & 0 & r_y \\ 0 & 1 & 0 \\ -r_y & 0 & 1 \end{pmatrix} \tag{2b}$$

$$H_z \approx \begin{pmatrix} 1 & -r_z & 0 \\ r_z & 1 & 0 \\ 0 & 0 & 1 \end{pmatrix} \tag{2c}$$

By multiplying these together, we can find a single linearized rotation matrix H:

$$H = H_x H_y H_z \approx \begin{pmatrix} 1 & -r_z & r_y \\ r_x r_y + r_z & 1 - r_x r_y r_z & -r_x \\ r_x r_z - r_y & r_x + r_y r_z & 1 \end{pmatrix} \tag{3}$$

This doesn't look like much of a rotation matrix, and it's certainly not linear. However, because we're working under the assumption that r_x, r_y, and r_z are all very small, any two (or three) multiplied together can be approximated as zero. With this in mind, the rotation matrix becomes:

$$H' = \begin{pmatrix} 1 & -r_z & r_y \\ r_z & 1 & -r_x \\ -r_y & r_x & 1 \end{pmatrix} \tag{4}$$

This looks much more like a rotation matrix, and is linear. Next, we consider a simple point-to-plane, mean-squared error-based cost function over a surface, where p_i is the point on our stationary surface, A, n_i is the normal vector at that point, and q_i is the matched point on the transformed surface, B. Surfaces A and B have m_A and m_B points on them, respectively:

$$C = \frac{1}{m_A} \sum_{i=1}^{m_A} \left((p_i - (Hq_i - t)) \cdot n_i \right)^2 \tag{5}$$

Note that minimizing this is equivalent to minimizing:

$$C = \sum_{i=1}^{m_A} ((p_i - (Hq_i - t)) \cdot n_i)^2 \tag{6}$$

If we substitute H' for H and expand the vector notation, we get:

$$C = \sum_{i=1}^{m_A} ((\begin{pmatrix} p_{i,x} \\ p_{i,y} \\ p_{i,z} \end{pmatrix} - (\begin{pmatrix} 1 & -r_z & r_y \\ r_z & 1 & -r_x \\ -r_y & r_x & 1 \end{pmatrix} \begin{pmatrix} q_{i,x} \\ q_{i,y} \\ q_{i,z} \end{pmatrix} - \begin{pmatrix} t_x \\ t_y \\ t_z \end{pmatrix})) \cdot \begin{pmatrix} n_{i,x} \\ n_{i,y} \\ n_{i,z} \end{pmatrix})^2 \tag{7}$$

If we multiply this through, we obtain the following:

$$\begin{aligned} C = \sum_{i=1}^{m_A} & ((p_{i,x} - q_{i,x} + r_z q_{i,y} - r_y q_{i,z} + t_x) * n_{i,x} \\ & + (p_{i,y} - q_{i,y} + r_x q_{i,z} - r_z q_{i,x} + t_y) * n_{i,y} \\ & + (p_{i,z} - q_{i,z} + r_y q_{i,x} - r_x q_{i,y} + t_z) * n_{i,z})^2 \end{aligned} \tag{8}$$

Next, we rearrange to group the transformation terms:

$$\begin{aligned} C = \sum_{i=1}^{m_A} & (r_x(n_{i,y}q_{i,z} - n_{i,z}q_{i,y}) + r_y(n_{i,z}q_{i,x} - n_{i,x}q_{i,z}) \\ & + r_z(n_{i,x}q_{i,y} - n_{i,y}q_{i,x}) + t_x n_{i,x} + t_y n_{i,y} + t_z n_{i,z} \\ & + (p_{i,x} - q_{i,x}) * n_{i,x} + (p_{i,y} - q_{i,y}) * n_{i,y} + (p_{i,z} - q_{i,z}) * n_{i,z})^2 \end{aligned} \tag{9}$$

Here, we note the cross product $c_i = n_i \times p_i = \{n_{i,y}q_{i,z} - n_{i,z}q_{i,y}, n_{i,z}q_{i,x} - n_{i,x}q_{i,z}, n_{i,x}q_{i,y} - n_{i,y}q_{i,x}\}^T$. We can substitute this into our equation:

$$\begin{aligned} C = \sum_{i=1}^{m_A} & (r_x c_{i,x} + r_y c_{i,y} + r_z c_{i,z} + t_x n_{i,x} + t_y n_{i,y} + t_z n_{i,z} \\ & + (p_{i,x} - q_{i,x}) * n_{i,x} + (p_{i,y} - q_{i,y}) * n_{i,y} + (p_{i,z} - q_{i,z}) * n_{i,z})^2 \end{aligned} \tag{10}$$

Written in vector notation, this is just:

$$C = \sum_{i=1}^{m_A} (r \cdot c_i + t \cdot n_i + (p_i - q_i) \cdot n_i)^2 \tag{11}$$

To solve our equation for r and t, we take the partial derivative of equation 11 with respect to each component of r and t:

$$\frac{\partial C}{\partial r_x} = \sum_{i=1}^{m_A} 2(r \cdot c_i + t \cdot n_i + (p_i - q_i) \cdot n_i) * c_{i,x} \tag{12a}$$

$$\frac{\partial C}{\partial r_y} = \sum_{i=1}^{m_A} 2(r \cdot c_i + t \cdot n_i + (p_i - q_i) \cdot n_i) * c_{i,y} \tag{12b}$$

$$\frac{\partial C}{\partial r_z} = \sum_{i=1}^{m_A} 2(r \cdot c_i + t \cdot n_i + (p_i - q_i) \cdot n_i) * c_{i,z} \tag{12c}$$

$$\frac{\partial C}{\partial t_x} = \sum_{i=1}^{m_A} 2(r \cdot c_i + t \cdot n_i + (p_i - q_i) \cdot n_i) * n_{i,x} \tag{12d}$$

$$\frac{\partial C}{\partial t_y} = \sum_{i=1}^{m_A} 2(r \cdot c_i + t \cdot n_i + (p_i - q_i) \cdot n_i) * n_{i,y} \tag{12e}$$

$$\frac{\partial C}{\partial t_z} = \sum_{i=1}^{m_A} 2(r \cdot c_i + t \cdot n_i + (p_i - q_i) \cdot n_i) * n_{i,z} \tag{12f}$$

Finally, to get our linear system, we set these equal to zero and move all known terms to the other side:

$$\sum_{i=1}^{m_A} (r \cdot c_i + t \cdot n_i) * c_{i,x} = \sum_{i=1}^{m_A} -((p_i - q_i) \cdot n_i) * c_{i,x} \tag{13a}$$

$$\sum_{i=1}^{m_A} (r \cdot c_i + t \cdot n_i) * c_{i,y} = \sum_{i=1}^{m_A} -((p_i - q_i) \cdot n_i) * c_{i,y} \tag{13b}$$

$$\sum_{i=1}^{m_A} (r \cdot c_i + t \cdot n_i) \cdot c_{i,z} = \sum_{i=1}^{m_A} -((p_i - q_i) \cdot n_i) * c_{i,z} \tag{13c}$$

$$\sum_{i=1}^{m_A} (r \cdot c_i + t \cdot n_i) * n_{i,x} = \sum_{i=1}^{m_A} -((p_i - q_i) \cdot n_i) * n_{i,x} \tag{13d}$$

$$\sum_{i=1}^{m_A} (r \cdot c_i + t \cdot n_i) * n_{i,y} = \sum_{i=1}^{m_A} -((p_i - q_i) \cdot n_i) * n_{i,y} \tag{13e}$$

$$\sum_{i=1}^{m_A} (r \cdot c_i + t \cdot n_i) * n_{i,z} = \sum_{i=1}^{m_A} -((p_i - q_i) \cdot n_i) * n_{i,z} \tag{13f}$$

When expanded, this becomes a simple 6×6 linear system:

$$\sum_{i=1}^{m_A} \begin{pmatrix} c_{i,x}c_{i,x} & c_{i,x}c_{i,y} & c_{i,x}c_{i,z} & c_{i,x}n_{i,x} & c_{i,x}n_{i,y} & c_{i,x}n_{i,z} \\ c_{i,y}c_{i,x} & c_{i,y}c_{i,y} & c_{i,y}c_{i,z} & c_{i,y}n_{i,x} & c_{i,y}n_{i,y} & c_{i,y}n_{i,z} \\ c_{i,z}c_{i,x} & c_{i,z}c_{i,y} & c_{i,z}c_{i,z} & c_{i,z}n_{i,x} & c_{i,z}n_{i,y} & c_{i,z}n_{i,z} \\ n_{i,x}c_{i,x} & n_{i,x}c_{i,y} & n_{i,x}c_{i,z} & n_{i,x}n_{i,x} & n_{i,x}n_{i,y} & n_{i,x}n_{i,z} \\ n_{i,y}c_{i,x} & n_{i,y}c_{i,y} & n_{i,y}c_{i,z} & n_{i,y}n_{i,x} & n_{i,y}n_{i,y} & n_{i,y}n_{i,z} \\ n_{i,z}c_{i,x} & n_{i,z}c_{i,y} & n_{i,z}c_{i,z} & n_{i,z}n_{i,x} & n_{i,z}n_{i,y} & n_{i,z}n_{i,z} \end{pmatrix} \begin{pmatrix} r_x \\ r_y \\ r_z \\ t_x \\ t_y \\ t_z \end{pmatrix} = \sum_{i=1}^{m_A} \begin{pmatrix} -((p_i - q_i) \cdot n_i) * c_{i,x} \\ -((p_i - q_i) \cdot n_i) * c_{i,y} \\ -((p_i - q_i) \cdot n_i) * c_{i,z} \\ -((p_i - q_i) \cdot n_i) * n_{i,x} \\ -((p_i - q_i) \cdot n_i) * n_{i,y} \\ -((p_i - q_i) \cdot n_i) * n_{i,z} \end{pmatrix} \tag{14}$$

Now consider the symmetric formulation. Instead of equation 6, where we only minimize the distance from one surface to another, we want a cost function that takes both surfaces into consideration and minimizes the sum distance between them simultaneously. To do this, we simply add a second half to our existing function that is conceptually based on moving our stationary surface, A, and the paired point on it, p_i, to our transformed one, B, and the reference point q_i. This accounts for all points on A and B, but leaves us with a problem: We would have two sets of transformation parameters to apply, which, when applied, would be transforming our surfaces to outdated locations. Rather than solving for the transform or actually moving our stationary surface, we rewrite the new half of the cost function in terms of a transform applied to surface B, add it to our previous cost function, and solve the entire system instead. In this formulation, l_i is the normal vector at the q_i, because it is the reference point, even though it isn't stationary. Also, rather than rotating, then translating point p_i, we can choose to first translate, then rotate in order to preserve the independence of the transformation parameters when we rewrite it in terms of q_i:

$$C = \sum_{i=1}^{m_B} ((q_i - H(p_i - t)) \cdot l_i)^2 \tag{15}$$

To rewrite this in terms of a transformation of surface B, we rotate with H^T then reverse the translation:

$$C = \sum_{i=1}^{m_B} ((H^T q_i + t) - p_i)) \cdot l_i)^2 \tag{16}$$

With explicit matrices and vectors, this is:

$$C = \sum_{i=1}^{m_B} (((\begin{pmatrix} 1 & r_z & -r_y \\ -r_z & 1 & r_x \\ r_y & -r_x & 1 \end{pmatrix} \begin{pmatrix} q_{i,x} \\ q_{i,y} \\ q_{i,z} \end{pmatrix} + \begin{pmatrix} t_x \\ t_y \\ t_z \end{pmatrix}) - \begin{pmatrix} p_{i,x} \\ p_{i,y} \\ p_{i,z} \end{pmatrix}) \cdot \begin{pmatrix} l_{i,x} \\ l_{i,y} \\ l_{i,z} \end{pmatrix})^2 \tag{17}$$

When expanded, this becomes:

$$\begin{aligned} C = \sum_{i=1}^{m_B} & ((q_{i,x} + r_z q_{i,y} - r_y q_{i,z} + t_x - p_{i,x}) * l_{i,x} \\ & + (-r_z q_{i,x} + q_{i,y} + r_x q_{i,z} + t_y - p_{i,y}) * l_{i,y} \\ & + (r_y q_{i,x} - r_x q_{i,y} + q_{i,z} + t_z - p_{i,z}) * l_{i,z})^2 \end{aligned} \tag{18}$$

We may once again group these by our transform parameters:

$$\begin{aligned} C = \sum_{i=1}^{m_B} & (r_x(q_{i,z} l_{i,y} - l_{i,z} q_{i,y}) + r_y(l_{i,z} q_{i,x} - l_{i,x} q_{i,z}) \\ & + r_z(l_{i,x} q_{i,y} - l_{i,y} q_{i,x}) + t_x l_{i,x} + t_y l_{i,y} + t_z l_{i,z} \\ & + (q_{i,x} - pi, x) * l_{i,x} + (q_{i,y} - pi, y) * l_{i,y} + (q_{i,z} - pi, z) * l_{i,z})^2 \end{aligned} \tag{19}$$

As before, we note that $d_i = l_i \times q_i = \{q_{i,z} l_{i,y} - l_{i,z} q_{i,y}, l_{i,z} q_{i,x} - l_{i,x} q_{i,z}, l_{i,x} q_{i,y} - l_{i,y} q_{i,x}\}^T$, so we can rewrite the cost function in vector format:

$$C = \sum_{i=1}^{m_B} (r \cdot d_i + t \cdot l_i + (q_i - p_i) \cdot l_i)^2 \tag{20}$$

This equation is nearly identical to equation 11. The only differences are the cross product and normal vector replacements, and perhaps most importantly, the reversed order of the difference between the two points (i.e., $q_i - p_i$ and vice versa). Otherwise, the two equations are the same. In fact, because the partial derivative does not depend on the $(q_i - p_i) \cdot l_i$ or $(p_i - q_i) \cdot n_i$ parts, as they are all known values, we can simply substitute d_i and l_i on the left hand side of our 6×6 matrix and replace the right hand side with $(q_i - p_i) \cdot l_i$. The additional cost for the symmetric superimposition is then:

$$
\sum_{i=1}^{m_B} \begin{pmatrix} d_{i,x}d_{i,x} & d_{i,x}d_{i,y} & d_{i,x}d_{i,z} & d_{i,x}l_{i,x} & d_{i,x}l_{i,y} & d_{i,x}l_{i,z} \\ d_{i,y}d_{i,x} & d_{i,y}d_{i,y} & d_{i,y}d_{i,z} & d_{i,y}l_{i,x} & d_{i,y}l_{i,y} & d_{i,y}l_{i,z} \\ d_{i,z}d_{i,x} & d_{i,z}d_{i,y} & d_{i,z}d_{i,z} & d_{i,z}l_{i,x} & d_{i,z}l_{i,y} & d_{i,z}l_{i,z} \\ l_{i,x}d_{i,x} & l_{i,x}d_{i,y} & l_{i,x}d_{i,z} & l_{i,x}l_{i,x} & l_{i,x}l_{i,y} & l_{i,x}l_{i,z} \\ l_{i,y}d_{i,x} & l_{i,y}d_{i,y} & l_{i,y}d_{i,z} & l_{i,y}l_{i,x} & l_{i,y}l_{i,y} & l_{i,y}l_{i,z} \\ l_{i,z}d_{i,x} & l_{i,z}d_{i,y} & l_{i,z}d_{i,z} & l_{i,z}l_{i,x} & l_{i,z}l_{i,y} & l_{i,z}l_{i,z} \end{pmatrix} \begin{pmatrix} r_x \\ r_y \\ r_z \\ t_x \\ t_y \\ t_z \end{pmatrix} = \sum_{i=1}^{m_B} \begin{pmatrix} ((p_i - q_i) \cdot l_i) * d_{i,x} \\ ((p_i - q_i) \cdot l_i) * d_{i,y} \\ ((p_i - q_i) \cdot l_i) * d_{i,z} \\ ((p_i - q_i) \cdot l_i) * l_{i,x} \\ ((p_i - q_i) \cdot l_i) * l_{i,y} \\ ((p_i - q_i) \cdot l_i) * l_{i,z} \end{pmatrix} \tag{21}
$$

The final linear system is just the sum of these two systems (equations 14 and 21). Once we solve them, we obtain an approximated minimizing transformation for the given correspondences. Because the matrices themselves are symmetric, the system can be easily solved with a Cholesky solver.

2 Simple Binary Space Partitioning Tree

For a detailed explanation, we refer the reader to Pettinger [1], but we provide here a basic explanation of the algorithm we implemented based on bisecting k-means.

First, we'll discuss k-means clustering. k-means clustering is an iterative algorithm aimed primarily at separating data into groups. We start with a collection of points in some space. We select some number of these points to be "generator" points. On the first iteration, we associate each point in our collection with the nearest generator to it. We then update the generator position to be the centroid of the group of points associated with it. On the next iteration, we repeat the association step of the algorithm and the subsequent generator-position update. Eventually, we will reach a relatively even spacing of the generators based on the distribution of points in our collection.

By placing this k-means clustering method inside a recursively branching parent-child structure, we can create a simple binary space partitioning tree. If we cluster our entire surface for only two generators at a time, we effectively split the surface into two "leaves". We then count how many points are in each of these leaves. If either is larger than some lower bound, we perform two-generator k-means clustering on the points in the leaf, creating two new leaves. The data stored in the old leaf, except for the position of its generator and pointers to the two new leaves, is removed, converting the old leaf into a branch. This process is repeated until no single leaf is larger than the lower bound. In practice, we found that fifty points per subsection was an adequate lower bound that did not introduce problematic cross-boundary nearest-neighbor mispairings that would otherwise potentially affect the superimposition speed or quality.

To traverse this tree for a nearest neighbor search, given a point p, we simply start at the first pair of generators, g_1 and g_2. We measure the distance to each, then pick the one that's closest to p. If the generator corresponds to a branch, with child-generators h_1 and h_2, we again check to see which is closer, and pick that one. The process is repeated until we reach a leaf, rather than a branch. Once we reach a leaf, we search through all the points in the leaf until we find the closest one to p.

This technique worked well when applied to our GPSA method as it was trivial to apply the rotation/translation/scale parameters to the network of nodes making up the tree. However, it also introduced a stochastic element to the method. Because the generators are selected at random, this can, in cases of extreme variation, somewhat alter the final mean surface. In practice, our single species data set had very little variation in the distribution of points in the final mean surfaces between testing runs, while the multi-species data set had a large amount of variation in point positions, but these points ultimately made up a relatively consistent surface. There was one primary exception to this, where neither the point distribution

nor the surfaces were conserved: The canines and incisors in the multi-species final mean surface occasionally had a single region that protruded from an otherwise smooth surface. This appears to be caused by a single terminating leaf in the tree becoming disjointed from the rest of the surface. In testing, this disjointed section occurred in four out of ten identical trial runs aimed at elucidating this issue.

3 Duplicate Point Elimination

One of the trickiest problems in the algorithm is the elimination of duplicated points from the prototype surface. A duplicate point is where two points on the prototype, p_1 and p_2, are both paired with the same set of points on sample surfaces $s_1, s_2, ..., s_m$. When averaged, these two points will end up in the same location, effectively doubling the weight of that single point on the prototype.

To avoid this, we have to search for and delete such duplicated points. Naively, we would simply store an array of point indices of matched points for each point on the prototype, then search through them all to verify there are no duplicates. Computationally speaking, this is very expensive, an O(mn^2) operation, where n is the number of points on the prototype and m is the number of specimen. We can bring this down to O(nm) at the expense of memory through use of indexing and pointers.

First, we'll define the way our surfaces are stored. A single surface, S, of n three dimensional points is simply a two dimensional array of floating point numbers of size nx3. Next, we'll define the two dimensional array of integers representing the point associations indices for the prototype, P_a, which has n_p rows and m columns. Row r would refer to point q_r on the prototype surface, column c would refer to sample surface S_c, and the integer stored there would refer to the row index of S_c that stores the nearest neighbor match to point q_r.

We need four more arrays, namely, a 1D array of integers, I_s and a 1D array of booleans, B, of length n_c where n_c is the number of points in sample surface S_c. We also need two 1D arrays of integers, $I_{p,new}$ and $I_{p,old}$ of length n_p where n_p is the number of points in the prototype surface. On the first iteration of the algorithm, we initialize the array of booleans all as false, then loop (using iterator i) over the first column of the point association array (corresponding to the first surface in the sample). For each entry in the column c_i, we check whether $B[c_i]$ is false, indicating that particular point hasn't been matched yet. If $B[c_i]$ is indeed false, we store i at $I_s[c_i]$ and at $I_{p,old}[i]$). If $B[c_i]$ is true, then we know that c_i has been paired to another point. In that case, we store the value of $I_{p,old}[I_s[c_i]]$ at $I_{p,old}[i]$.

On the second iteration, we do almost the same thing: We initialize the array of booleans all as false, then loop (using iterator i) over the second column of the point association array. For each entry in the column c_i, we check whether $B[c_i]$ is false. If $B[c_i]$ is false, we store i at $I_s[c_i]$ and at $I_{p,new}[i]$. If $B[c_i]$ is true, then we check to see if $I_{p,old}[c_i]$ is the same as $I_{p,old}[i]$. If it is, it means that not only are the point correspondences the same for this surface for these two particular points, they were also the same for the last surface. In this case, we set $I_{p,new}[i]$ to the same value as $I_{p,new}[I_s[c_i]]$. Otherwise, we set $I_{p,new}[i]$ to i, because even though the points have been the same on the previous iteration, they are now distinct. After

this iteration, we can set $I_{p,old} = I_{p,new}$. In doing this, we know that if two entries in $I_{p,new}$ have the same index, then they had the same point associations up to that iteration.

On the final iteration, rather than storing index entries in $I_{p,new}$, we can simply store '1' or '0', indicating whether the point should be kept or thrown away. If the point associations have been the same up to this point, then they are identical if they are the same on this final iteration. This array can be looped over a final time for the actual removal of duplicates from the prototype surface data array.

4 Ordination and Dimensional Reduction

In landmark-based geometric morphometrics (e.g., Rohlf and Slice, 1990 [7]), homologous landmarks are translated, scaled, and rotated to optimum alignment to an iteratively computed mean shape so that the resulting coordinates can be subjected to multivariate statistical analysis. These analyses include the computation of descriptive statistics, such as mean shapes and covariance structure, comparison of group means, covariation of shape with external variables like temperature or altitude, etc. Multivariate texts, such as Johnson and Wichern (2007) [4] and Krzanowski (2000) [5], outline the numerous analyses available. Many of these methods, however, are dependent upon a data covariance matrix of full rank, i.e., that is invertible. The constraints imposed by the superimposition process, however, guarantee a singular covariance matrix. This problem can be addressed, for instance, by projection of the data onto the partial or relative warps (Rohlf and Bookstein, 2003 [6]) of appropriate dimension, as is done in Rohlf's tps series of programs (e.g., Rohlf 2014 [8]).

The use of partial or relative warp scores does not entirely solve the problem of covariance singularity for many data sets, especially in geometric morphometrics. For a data set of n objects and p variables, there are at most $min(n-1, p)$ non-zero eigenvalues. Hence, when p exceeds $(n-1)$, the covariance matrix is guaranteed to be singular (total eigenvalues exceed number of non-zero ones). The most common means of addressing this situation is through dimension reduction through ordination, and this is usually by principal components analysis (PCA), where the data are projected onto a subset of the eigenvectors associated with non-zero eigenvalues of the covariance matrix prior to extended multivariate analysis. This process usually involves the extraction of the eigenvectors/values of the $p \times p$ covariance matrix.

Even data reduction through PCA, though, might be insufficient for practical application in a GPSA analysis due to the extreme number of variables involved - the three coordinates of all of the homologized surface vertices. The data matrix for the mixed-species sample after GPSA in our example, for instance, is 18×2203611, and the same for the single-species, single-sex sample is 15×1561422. A PCA of these would involve the extraction of the eigenvectors of covariance matrices of 2203611×2203611 and 1561422×1561422, respectively, which could be prohibitively time consuming. Gower (1966) [3], however, provided a method for constructing the same ordination plots (up to reflection) using the inter-specimen distance matrix. In the present case, this only requires extracting the eigenvectors/values of an 18×18 or 15×15 matrix. The limiting aspect of Gower's Principal Coordinates Analysis, PCOORD, is that the eigenvectors directly represent coordinates in PCOORD/PCA space and do not provide the weightings for the individual variables that are used for the construction of visualizations in the normal GM-PCA approach. To address these, we

regress the homologized vertex coordinates of individual specimens onto their scores on the PCOORD axes. This still involves rather large matrices, but avoids the construction and decomposition of vertex-coordinate covariance matrices required by PCA.

The summary results of the above procedure applied to the mixed-species sample are shown in Table 1, which gives the variances of the projections of the specimens on the PCOORD axes, the proportion of variance accounted for by each axis, and the cumulative proportion of variance accounted for by the current and all axes associated with greater variation. Variances are plotted in the top of Figure 1, and indicate a rather familiar pattern of the first few (one in this case) axes accounting for a larger proportion of variation with variance on higher-numbered axes trailing off gradually. As shown in Figure 8 in the article text, this represents a contrast between the very different morphologies of the specimens of the genus Papio with the other specimens.

Figure 2 (top) shows renderings of the actual specimens representing extremes of variation on the first axis, and below are the visualizations of reconstructions based only on this axis. Vertices are colored according to the root sum-of-squared regression coefficient to emphasize regions most associated with this regression. For this axis, there is clearly an emphasis on the brow-ridge area, and the method appears to have difficulty homologizing vertices of the orbit in this extreme case. We refrain from further interpretation since this data set is intended as a "worst-case" example for illustrative purposes only.

Figure 3 shows the same renderings for the second-greatest axis of variation for the mixed-species sample. Differences are much less dramatic as indicated by the total variance for this axis in Table 1, but some emphasis is placed on the temporal region and zygomatic arch (orange-red coloring). Note that the specimen with the lowest score on this axis, specimen 6, genus Chiropotes, is the same specimen having the highest projection on the first axis.

Table 2 shows the summary results for the analysis of the single-species, single-sex sample, and Figure 4 plots the variance versus axis number. In this much more homogeneous sample (by design), variance declines much more gradually with increasing axis number. The specimens with the lowest and highest projections on the first axis are shown in Figure 5 (top) along with the visualizations of their projections (bottom). This axis emphasizes variation in the bulbousness behind the brow ridge, but also picks up some high variability in the back of the orbit and within the nasal aperture. The latter is likely due to problems in homologizing poorly scanned, partially occluded regions. As mentioned in the article, data were not cleaned for this exposition. More careful editing of the specimen and prototype configurations could well address this issue in research applications.

Figure 6 shows the same renderings and visualizations for the axis of second greatest variation. Patterns shown here are subtle, though there is some indication of difficulties in homology mapping in semi-occluded surfaces in the orbit and nasal areas as seen in the previous figure.

MIXED SAMPLE

Rank	Variance	Proportion	Cumulative Proportion
1	1237.04	0.47	0.47
2	323.76	0.12	0.6
3	188.79	0.07	0.67
4	134.95	0.05	0.72
5	116.78	0.04	0.76
6	97.08	0.04	0.8
7	75.98	0.03	0.83
8	67.94	0.03	0.86
9	58.74	0.02	0.88
10	51.33	0.02	0.9
11	49.13	0.02	0.92
12	45.24	0.02	0.93
13	41.97	0.02	0.95
14	36.85	0.01	0.96
15	33.53	0.01	0.98
16	31.01	0.01	0.99
17	28.94	0.01	1

Table 1: **Summary statistics for variance-based ordination (PCOORD/PCA) of the mixed-species sample used in this paper.** Column one shows axes ranked by variance. Column two shows the variance on that axis. Columns three and four shows the proportion of variance associated with a particular axis and the cumulative variances for that and all higher-variance axes.

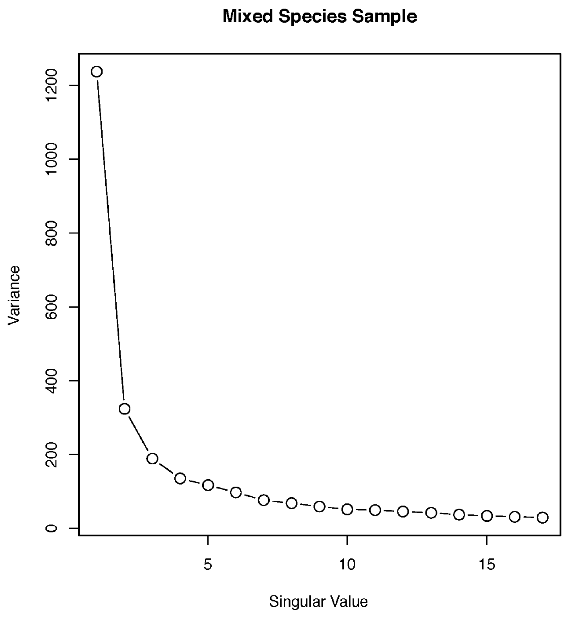

Figure 1: **Plot of PCOORD/PCA axes of the mixed-species sample sorted by the variance associated with them.**

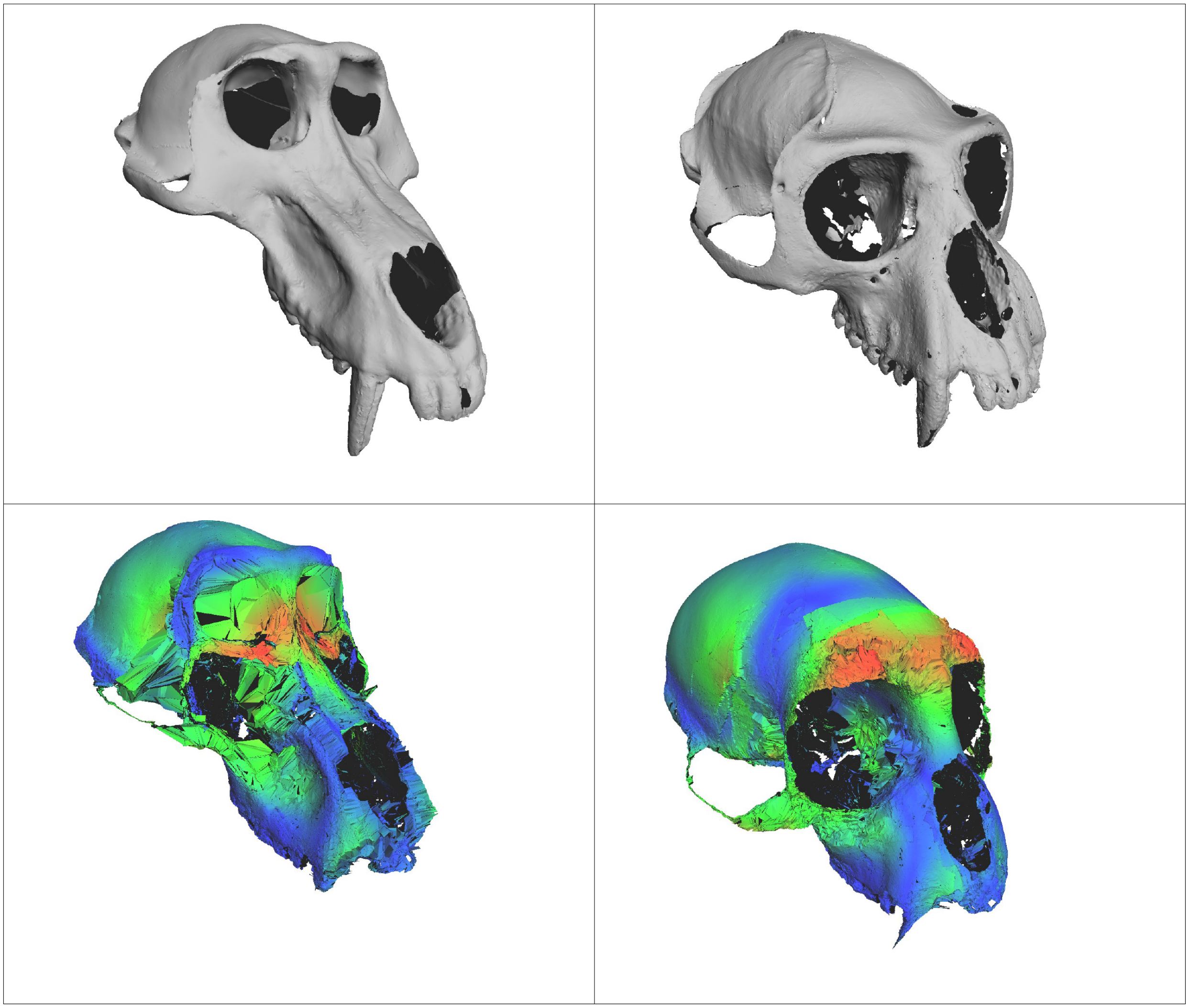

Figure 2: **Renderings of specimens from the mixed-species sample with extreme projections on the first PCOORD/PCA axis (top) and their approximations based on that axis (bottom).** Approximations based on linear regression of all specimen surfaces on their projections onto this axis. Coloring of the approximations based on the root sum of squared regression coefficients for each coordinate of a vertex. Colors linearly mapped from lowest to highest: B $\to$ G $\to$ R. See text for details.

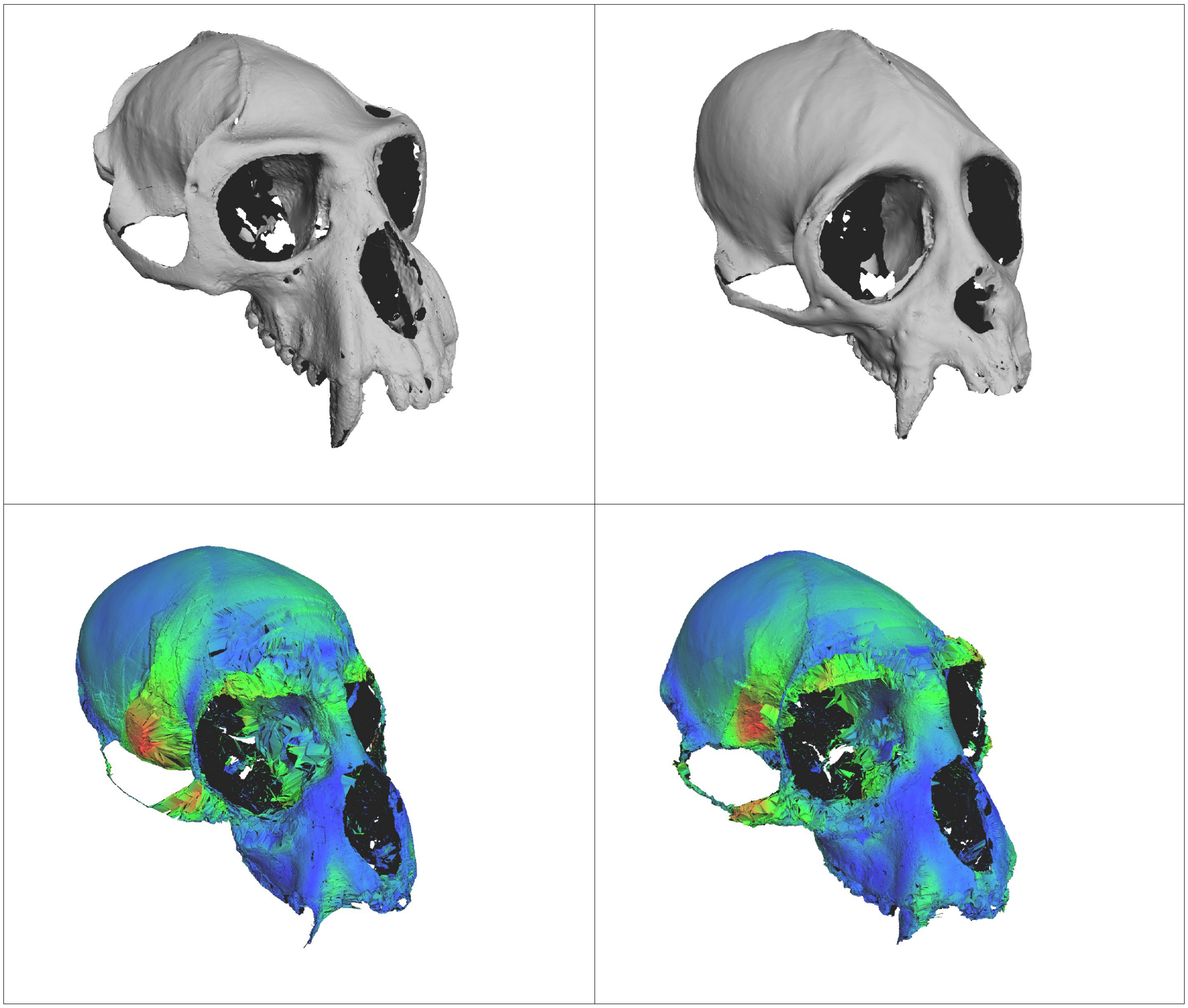

Figure 3: **Renderings of specimens from the mixed-species sample with extreme projections on the second PCOORD/PCA axis (top) and their approximations based on that axis (bottom).** Approximations based on linear regression of all specimen surfaces on their projections onto this axis. Coloring of the approximations based on the root sum of squared regression coefficients for each coordinate of a vertex. Colors linearly mapped from lowest to highest: B $\to$ G $\to$ R. See text for details.

SINGLE SPECIES SAMPLE

Rank	Variance	Proportion	Cumulative Proportion
1	59.39	0.19	0.19
2	40.07	0.13	0.32
3	33.79	0.11	0.42
4	29.61	0.09	0.52
5	22.91	0.07	0.59
6	22.24	0.07	0.66
7	19.72	0.06	0.73
8	15.72	0.05	0.78
9	13.9	0.04	0.82
10	13.2	0.04	0.86
11	12.07	0.04	0.9
12	11.5	0.04	0.94
13	10.42	0.03	0.97
14	9.46	0.03	1

Table 2: **Summary statistics for variance-based ordination (PCOORD/PCA) of the single-species, single-sex sample used in this paper.** Column one shows axes ranked by variance. Column two shows the variance on that axis. Columns three and four shows the proportion of variance associated with a particular axis and the cumulative variances for that and all higher-variance axes.

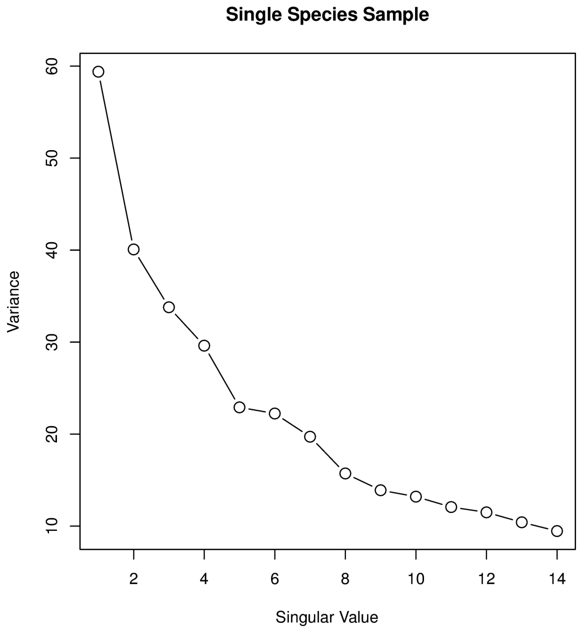

Figure 4: **Plot of PCOORD/PCA axes of the single-species, single-sex sample sorted by the variance associated with them.**

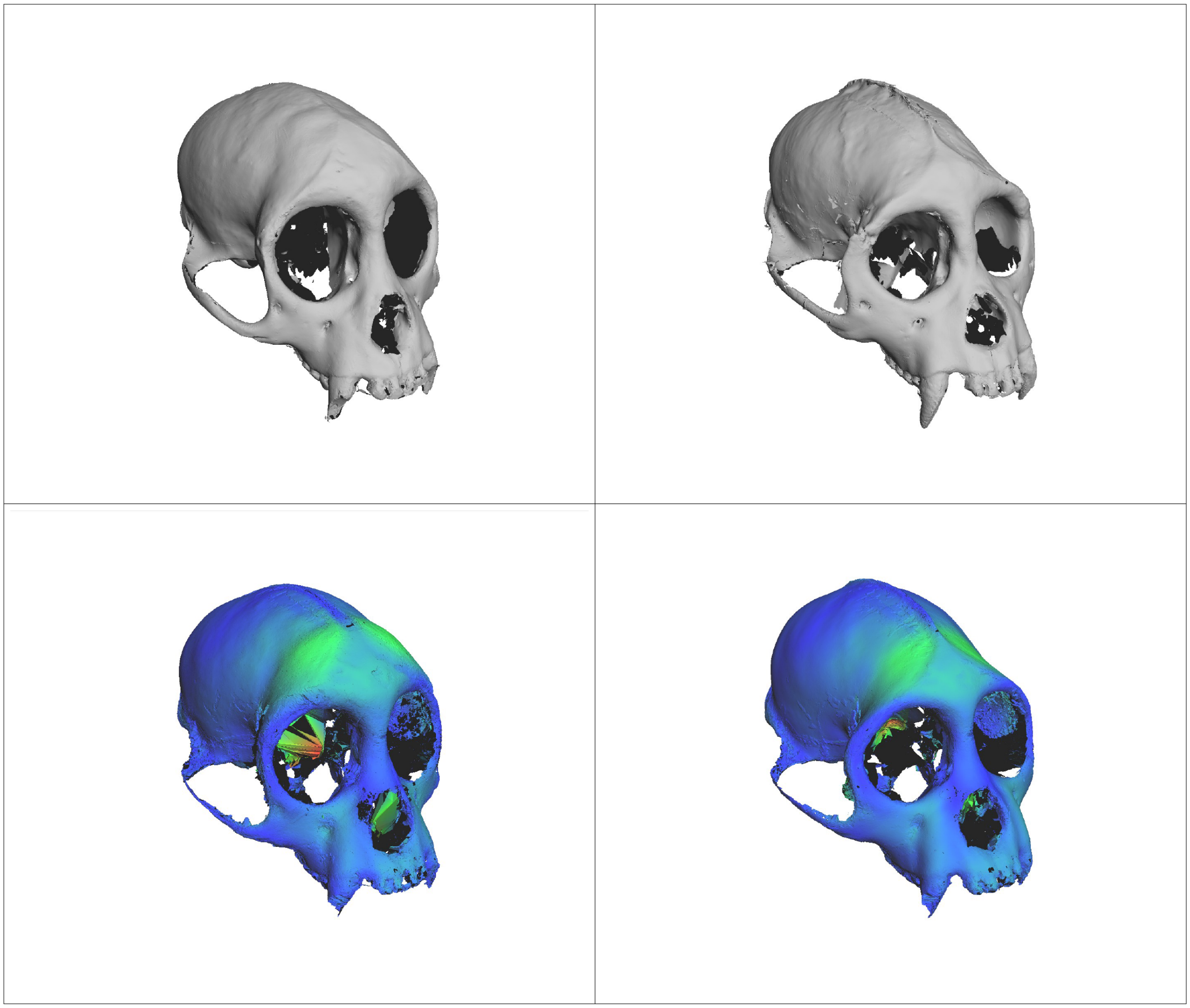

Figure 5: **Renderings of specimens from the single-species, single-sex sample with extreme projections on the first PCOORD/PCA axis (top) and their approximations based on that axis (bottom).** Approximations based on linear regression of all specimen surfaces on their projections onto this axis. Coloring of the approximations based on the root sum of squared regression coefficients for each coordinate of a vertex. Colors linearly mapped from lowest to highest: B $\rightarrow$ G $\rightarrow$ R. See text for details.

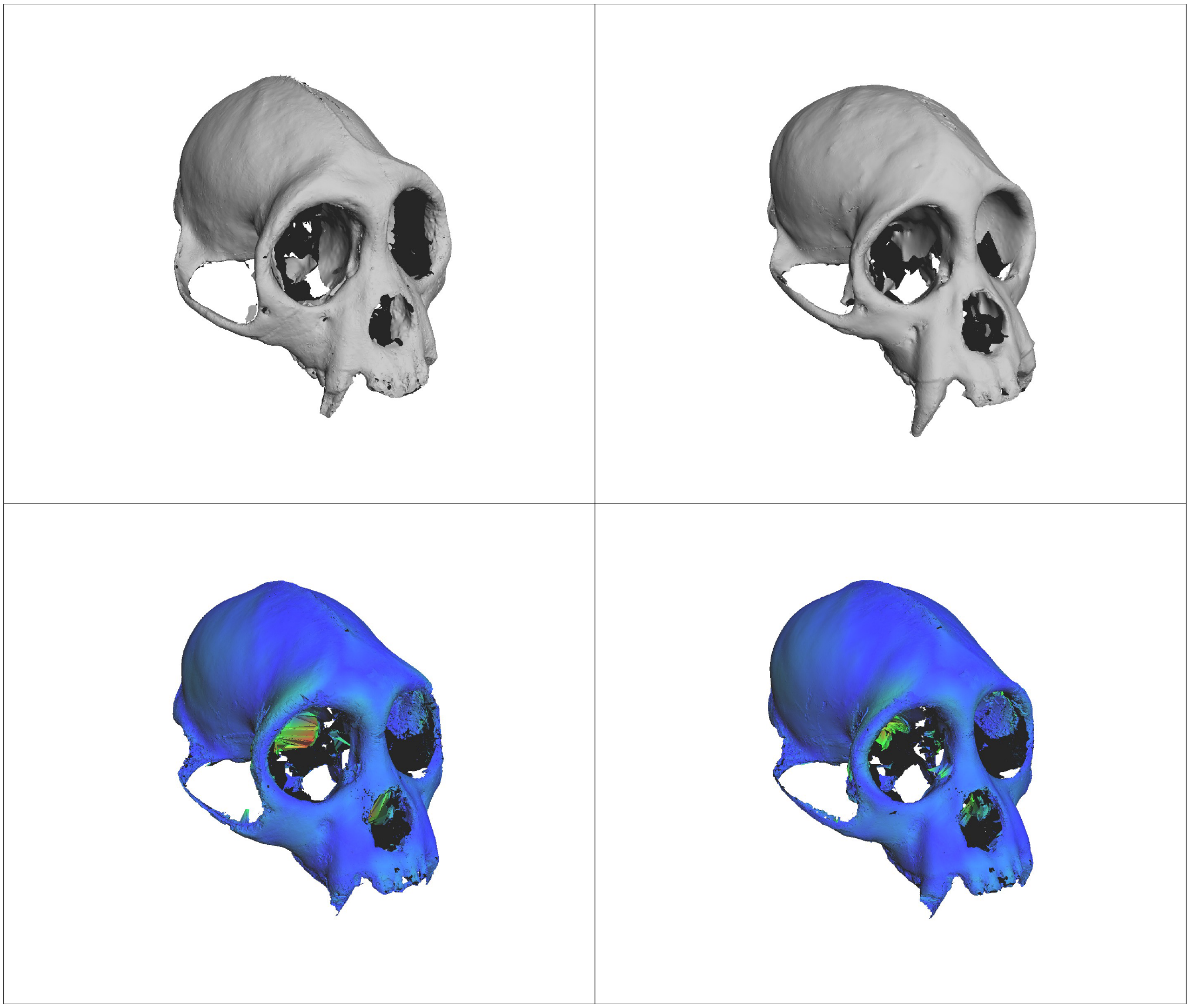

Figure 6: **Renderings of specimens from the single-species sample with extreme projections on the second PCOORD/PCA axis (top) and their approximations based on that axis (bottom).** Approximations based on linear regression of all specimen surfaces on their projections onto this axis. Coloring of the approximations based on the root sum of squared regression coefficients for each coordinate of a vertex.Colors linearly mapped from lowest to highest: B $\rightarrow$ G $\rightarrow$ R. See text for details.

5 Erratum

Initial work on the GPSA method was developed in Pomidor (2013) [9]. In that work, a strong banding pattern appeared in the interspecimen distance plots comparing GPSA and GPA using the Procrustes Surface Metric. In preparing this paper, it was found that the BSP tree was not updated correctly when calculating PSM values for imported GPA superimpositions, which appears to have been the source of the original banding pattern. It should be noted that this issue only affected cases where external GPA superimposition transformation parameters were loaded and applied to a surface that was subsequently used to calculate PSM values. This has been corrected and all results reported here are based on the corrected code.

References

[1] Pettinger D, Di Fatta G. Space Partitioning for Scalable K-Means. In: 2010 Ninth International Conference on Machine Learning and Applications (ICMLA); 2010. p. 319–324.

[2] Rusinkiewicz Szymon. Unpublished ICP Stability paper 2003.

[3] Gower JC. Some Distance Properties of Latent Root and Vector Methods Used in Multivariate Analysis. BIOMETRIKA 53; 1966. p. 325–38.

[4] Johnson Richard A., Wichern Dean W.. Applied Multivariate Statistical Analysis. Englewood Cliffs, N.J: Prentice-Hall, 2007.

[5] Krzanowski, W. J.. Principles of Multivariate Analysis: A Users Perspective. Oxford University Press; 2000.

[6] Rohlf FJ, Bookstein FL. Computing the Uniform Component of Shape Variation. SYSTEMATIC BIOLOGY 52, no. 1 ; 2003. p. 66–99.

[7] Rohlf FJ, Slice D EXTENSIONS OF THE PROCRUSTES METHOD FOR THE OPTIMAL SUPERIMPOSITION OF LANDMARKS. SYSTEMATIC ZOOLOGY 39, no. 1 ; 1990. p. 40–59.

[8] Rohlf FJ tpsRelw: Relative warps. Department of Ecology and Evolution, SUNY at Stony Brook. http://life.bio.sunysb.edu/morph/; 2014.

[9] Pomidor B GENERALIZED PROCRUSTES SURFACE ANALYSIS: A LANDMARK-FREE APPROACH TO SUPERIMPOSITION AND SHAPE ANALYSIS (Master's thesis). (2013). Florida State University.
